# TUFT1 interacts with RABGAP1 and regulates mTORC1 signaling

**DOI:** 10.1038/s41421-017-0001-2

**Published:** 2018-01-09

**Authors:** Natsumi Kawasaki, Kazunobu Isogaya, Shingo Dan, Takao Yamori, Hiroshi Takano, Ryoji Yao, Yasuyuki Morishita, Luna Taguchi, Masato Morikawa, Carl-Henrik Heldin, Tetsuo Noda, Shogo Ehata, Kohei Miyazono, Daizo Koinuma

**Affiliations:** 1 Department of Molecular Pathology, Graduate School of Medicine, The University of Tokyo, Tokyo, 113-0033 Japan; 2 Division of Molecular Pharmacology, Cancer Chemotherapy Center, Japanese Foundation for Cancer Research, Tokyo, 135-8550 Japan; 3 Division of Cell Biology and Director’s Room, Cancer Institute, Japanese Foundation for Cancer Research, Tokyo, 113-0033 Japan; 4 Ludwig Institute for Cancer Research, Science for Life Laboratory, Uppsala University, SE-751 24 Uppsala, Sweden; 5 Department of Medical Biochemistry and Microbiology, Science for Life Laboratory, Box 582, Uppsala University, SE-751 23 Uppsala, Sweden; 6 Present Address: Center for Product Evaluation, Pharmaceuticals and Medical Device Agency, Tokyo, 100-0013 Japan

## Abstract

The mammalian target of rapamycin (mTOR) pathway is commonly activated in human cancers. The activity of mTOR complex 1 (mTORC1) signaling is supported by the intracellular positioning of cellular compartments and vesicle trafficking, regulated by Rab GTPases. Here we showed that tuftelin 1 (TUFT1) was involved in the activation of mTORC1 through modulating the Rab GTPase-regulated process. TUFT1 promoted tumor growth and metastasis. Consistently, the expression of TUFT1 correlated with poor prognosis in lung, breast and gastric cancers. Mechanistically, TUFT1 physically interacted with RABGAP1, thereby modulating intracellular lysosomal positioning and vesicular trafficking, and promoted mTORC1 signaling. In addition, expression of *TUFT1* predicted sensitivity to perifosine, an alkylphospholipid that alters the composition of lipid rafts. Perifosine treatment altered the positioning and trafficking of cellular compartments to inhibit mTORC1. Our observations indicate that TUFT1 is a key regulator of the mTORC1 pathway and suggest that it is a promising therapeutic target or a biomarker for tumor progression.

## Introduction

Regulation of intracellular compartment positioning and vesicular trafficking is essential for multiple biological processes. Rab GTPases play critical roles as master regulators in cellular compartment positioning and vesicular trafficking^1, 2^. Over 60 *RAB* genes are encoded in the human genome. Rab GTPases function as molecular switches through their guanine nucleotide-binding status, like the other Ras superfamily proteins. Many Rab proteins are involved in cancer progression. For example, increased abundance of Rab5A and expression of *RAB7* occur in breast and lung cancer, respectively^3, 4^. *RAB25* is frequently amplified in breast and ovarian cancers and is associated with poor prognosis^5^. However, the mechanisms by which dysregulated expression of Rab GTPases contribute to tumorigenesis are poorly understood.

mTOR is a serine-threonine kinase that regulates cell growth and survival; its deregulation is frequently observed in human diseases, including type II diabetes and cancer^6, 7^. mTOR is an attractive target for cancer therapy, because the activation of phosphoinositide 3-kinase (PI3K)-mTOR signaling promotes resistance to conventional chemotherapies. mTOR forms two distinct multiprotein complexes, complex 1 (mTORC1) and complex 2 (mTORC2)^8–10^. In response to various physiological factors, including growth factors, energy status and amino acids, mTORC1 regulates multiple types of cellular processes, including mRNA translation. The small GTPase Rheb directly regulates mTORC1 activation downstream of the PI3K and AMP-activated protein kinase (AMPK) pathways and is activated by growth factors and glucose^11^. Amino acid-dependent mTORC1 activation requires four Rag family small GTPases: RagA, RagB, RagC, and RagD^12, 13^. The nucleotide-binding states of Rag complexes control the intracellular relocalization of mTORC1 from the cytosol to the lysosomal surface in response to amino acids. mTORC1 is then directly activated by GTP-bound Rheb GTPase on the surface of the lysosome^14^.

Intracellular compartment positioning and vesicular trafficking determine the activity of mTORC1 signaling, in particular, through lysosomal localization^15, 16^. In a cellular model of Huntington’s disease, perinuclear accumulation of lysosomes and mTORC1 hyper-activation are observed. In addition, overexpression of GDP- or GTP-bound mutants of several Rab GTPases strongly inhibits mTORC1 activation^17^. These results indicate that cycling and cellular trafficking of Rab GTPases are required for mTORC1 activation. However, the regulatory proteins that mediate the vesicular trafficking in the context of mTORC1 remain to be characterized.

Tuftelin 1 (TUFT1) is evolutionally conserved and is thought to play a role in the mineralization of dental enamel, which covers vertebrate teeth^18, 19^. However, TUFT1 is also found in non-mineralizing tissues and in various tumors^20, 21^. In the pheochromocytoma cell line PC12, TUFT1 abundance is increased by hypoxia in a hypoxia-inducible factor-1α-dependent manner^22, 23^. TUFT1 is therefore considered to be involved in cancer, but its physiological functions in normal and cancerous tissues remain uncharacterized.

Here, we demonstrate that TUFT1 is a key regulator of the mTORC1 signaling pathway. TUFT1 deficiency caused dispersion of the transport vesicles and lysosomes, and inhibition of mTORC1 signaling. We determined that TUFT1 promoted perinuclear lysosomal accumulation and intracellular vesicular trafficking by binding to RABGAP1, a GAP for certain Rab GTPases. Our investigations also highlighted the importance of TUFT1 in tumor growth and metastasis both in vitro and in vivo. We also revealed that sensitivity to perifosine, an alkylphospholipid AKT inhibitor, strongly correlated with expression of *TUFT1*. Unlike other PI3K-AKT inhibitors, perifosine acts at lipid rafts and inhibited lysosomal accumulation and mTORC1 signaling. These findings implicate that TUFT1 could be a promising therapeutic target or a biomarker for tumor progression.

## Results

### TUFT1 is a poor prognostic factor in various cancers

**Fig. 1:**
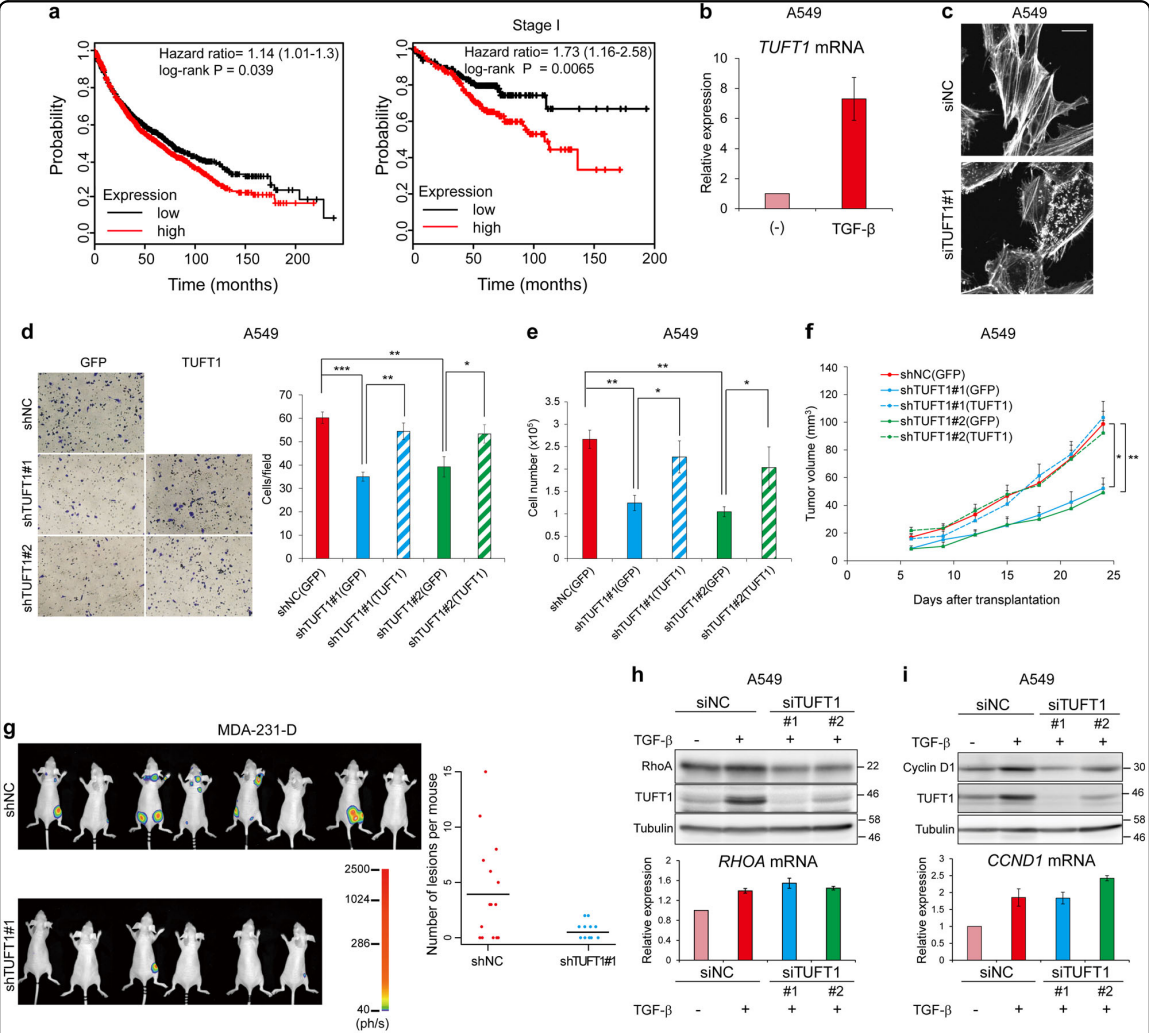
TUFT1, a TGF-β-induced poor cancer prognostic factor, regulates tumor cell morphology, motility and proliferation. **a** Kaplan Meier plot of overall survival stratified by *TUFT1* expression using the KM-Plotter version 2015 of lung cancer meta-analysis data. In the left panel, probability of overall survival of 1,926 patients split by median was displayed. In the right panel, stage I lung adenocarcinoma cases were subjected to the same analysis. **b** qRT-PCR analysis of A549 cells to evaluate the responsiveness of *TUFT1* mRNA expression to TGF-β (1 ng/mL) for 48 h. Results are means ± s.e.m. of three independent experiments. **c** A549 cells transfected with the indicated siRNAs were treated for 48 h with TGF-β and stained with fluorescein-conjugated phalloidin to visualize actin stress fibers. Images are representative of three independent experiments. Scale bar, 20 μm. **d** A549 cells expressing indicated shRNAs and proteins were seeded in upper chambers with collagen-coated pores. Exogenously expressed proteins are shown in parentheses. Cells that migrated through the membrane were counted. Representative fields are shown (left). Results are means ± s.e.m. of six independent experiments (right). *P < 0.05; **P < 0.01; ***P < 0.001. **e** A549 cells expressing indicated shRNAs and proteins (5 × 10^4^ cells) were seeded and cultured for 48 h. The live cell number of A549 cells was assessed by trypan blue staining. Exogenously expressed proteins are shown in parentheses. Data are presented as mean ± s.d. of three independent experiments. *P < 0.05; **P < 0.01. **f** A total of 1×10^6^ infected A549 cells expressing indicated shRNAs and proteins were xenografted and the volumes of the resulting tumors were measured. Exogenously expressed proteins are shown in parentheses. The result shows the combined data of the two independent experiments. Data are shown as mean ± s.e.m. *N* = 9 (shTUFT1#2 (GFP)) or 10 (others) mice per group. *P < 0.05; **P < 0.01. **g** Metastatic cells were analyzed by in vivo imaging in nude mice injected with MDA-231-D cells (1×10^5^ cells) expressing the indicated shRNAs. The horizontal bars indicate the mean for each group. *N* = 15 mice for the shNC group, *N* = 11 mice for the shTUFT1#1 group. The experiment was repeated with similar results, and representative images are shown (left). Dot plot for number of lesions per mouse from the two independent experiments is shown in the right panel. ph/s: photon counts per second. **h** A549 cells were transfected with the indicated siRNAs and treated with or without TGF-β for 48 h. Total amounts of RhoA were detected by immunoblotting. Results are representative of three independent experiments. mRNA expression was quantified by qRT-PCR (bottom panel). Results are means ± s.e.m. of three independent experiments. **i** Cells were cultured as in **h**. Cell lysates were analyzed by immunoblotting. Results are representative of two independent experiments. mRNA expression of cyclin D1 (*CCND1*) was quantified by qRT-PCR (bottom panel). Results are means ± s.e.m. of three independent experiments.

Thyroid transcription factor-1 (TTF-1, also known as NKX2-1), which is mainly found in thyroid and lung, is a prognostic indicator of non-small-cell lung cancer^24^. By analyzing data from chromatin immunoprecipitation-based sequencing using antibodies against TTF-1 and SMAD3^25^, we identified TUFT1 as a direct target of transforming growth factor β (TGF-β) which was inhibited by TTF-1 in NCI-H441 lung adenocarcinoma cells. Based on a public meta-analysis data, we found that high *TUFT1* expression was correlated with poor prognosis in lung (Fig. 1a), breast (Supplementary Figure S1A) and gastric cancer (Supplementary Figure S1B) patients^26, 27^. Particularly in the patients with stage I lung adenocarcinoma, TUFT1 expression was more significantly associated with poor prognosis (Fig. 1a).

We observed high expression of *TUFT1* in TTF-1-negative A549 lung adenocarcinoma cells compared to TTF-1-positive NCI-H441 cells (Supplementary Figure S1C); furthermore, TUFT1 expression was strongly induced by TGF-β and the protein was located mainly in the cytoplasm (Fig. 1b and Supplementary Figure S1D). To determine whether the induction of *TUFT1* by TGF-β is Smad-dependent, we depleted Smad4 in A549 cells and examined mRNA expression. Knockdown of Smad4 inhibited the expression of *TUFT1*, suggesting that TGF-β stimulates the expression of *TUFT1* in a Smad-dependent manner (Supplementary Figure S1E). Phalloidin staining revealed morphological change in TUFT1-silenced cells (Fig. 1c). TGF-β promotes epithelial-mesenchymal transition (EMT) which is accompanied by increased invasiveness of cancer cells, including A549 cells^28^. However, the expression of the EMT markers *CDH1* (which encodes E-cadherin), *SNAI1* (which encodes SNAIL), and *FN1* (which encodes fibronectin) were not markedly affected by TUFT1 knockdown (Supplementary Figure S1F), suggesting that TUFT1 regulates cellular morphology in an EMT-independent manner.

Knockdown of TUFT1 decreased the motility of A549 cells (Fig. 1d). In addition, analysis of the single cell tracking experiment revealed that knockdown of TUFT1 decreased the average migration speed of these cells (Supplementary Figure S1G). TUFT1 depletion also inhibited cell proliferation (Fig. 1e), whereas knockdown of TUFT1 did not significantly affect the viability of A549 cells (Supplementary Figure S1H). The inhibition of cell motility and proliferation caused by knockdown of TUFT1 was rescued by forced expression of wild-type TUFT1 (Fig. 1d, e). When A549 cells were xenografted onto nude mice, knockdown of TUFT1 decreased the tumor volume, and it was rescued by wild-type TUFT1 (Fig. 1f). To further investigate whether TUFT1 also affects tumor metastasis, nude mice were intracardially injected with MDA-231-D-luc cells^29^. Bone metastatic sites were fewer in number in mice that received MDA-231-D-luc cells with TUFT1 knockdown (Fig. 1g), while forced expression of TUFT1 canceled the effect (Supplementary Figure S1I).

Members of the Rho family of small GTPases regulate subcellular cytoskeletal actin dynamics and cellular motility^30^. The total amounts of RhoA, Rac1 and Cdc42 proteins were decreased in A549 cells (Fig. 1h and Supplementary Figure S2A and B), and also in MDA-231-D cells to some extents (Supplementary Figure S2C and D), upon TUFT1 knockdown without decreases in the mRNA abundance. Exposure to the proteasome inhibitor MG132 did not restore the abundance of RhoA and Rac1 (Supplementary Figure S2E), indicating that the decrease in the abundance of these proteins was not due to enhanced proteasomal degradation. In addition, knockdown of TUFT1 led to a decrease in the abundance of cyclin D1 and cyclin D3, which act as cell cycle regulatory switch in proliferating cells, without decreasing the mRNA expression (Fig. 1i and Supplementary Figure S2F).

We then performed RNA sequencing to comprehensively investigate the effects of knockdown of TUFT1. The gene set enrichment analysis (GSEA) identified several significantly enriched gene ontology gene sets in the genes up-regulated in control siRNA-transfected cells, including mTORC1 signaling (Supplementary Figure S2G). Because cyclin D1, cyclin D3^31^, and Rho family members of small GTPases^32^ are major targets of mTORC1 at the translational level, we next focused on mTORC1 signaling.

### TUFT1 affects perinuclear accumulation of mTORC1 and the lysosomes

**Fig. 2:**
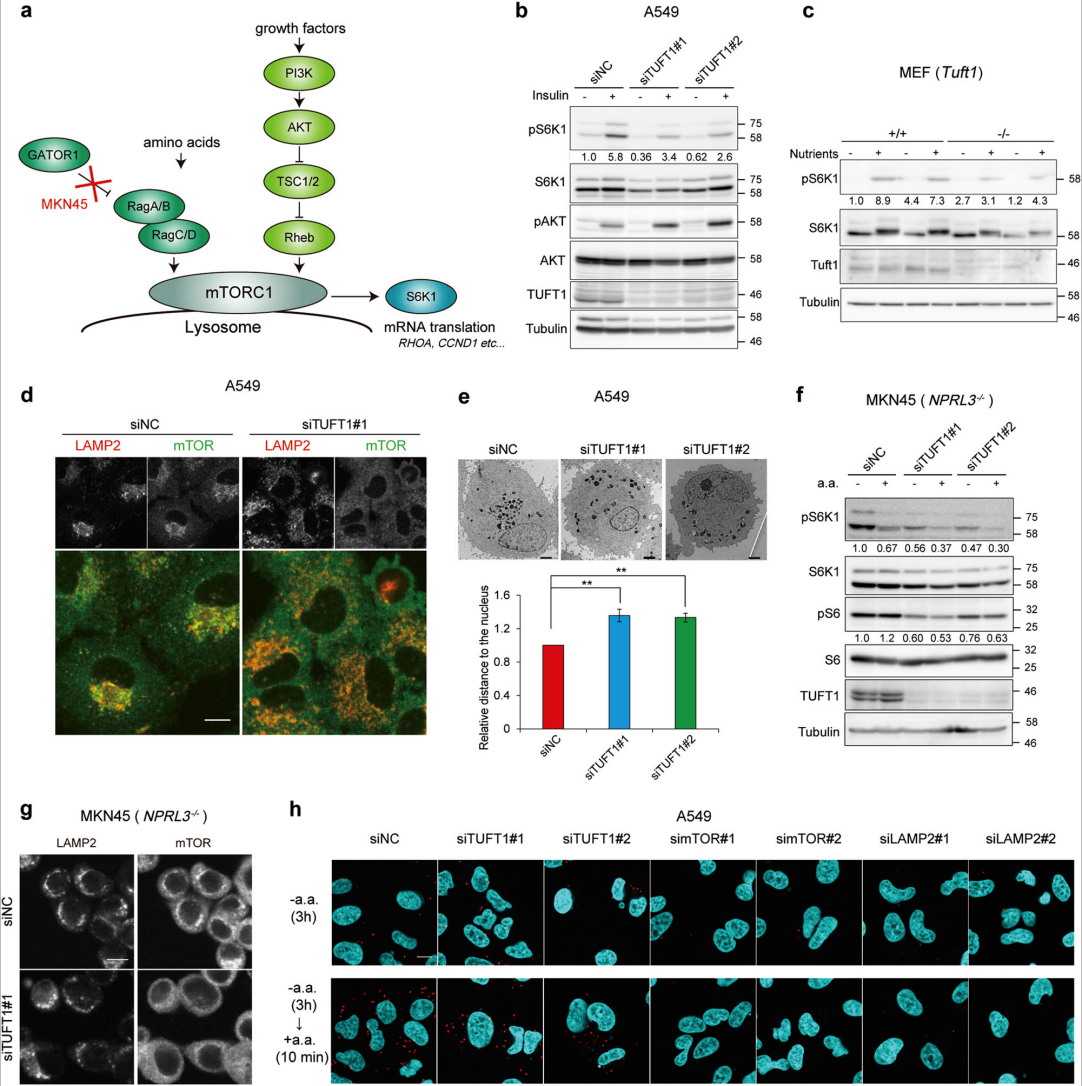
TUFT1 controls mTORC1 signaling and affects lysosomal positioning. **a** Schematic model of the mTORC1 activation pathway. **b** A549 cells treated with indicated siRNAs were serum starved for 24 h before stimulation with or without insulin (10 µg/mL) for 3 h. Cell lysates were analyzed by immunoblotting. Values in the panel show the amount of phosphorylated protein relative to the total amount of the protein, which were quantified by ImageJ. Results are representative of three independent experiments. **c** Cell lysates of wild type (+/+) and *Tuft1*^−/−^ MEFs (2 clones for each genotype) were starved (3 h) or starved and restimulated (10 min) with nutrients. Cell lysates were analyzed by immunoblotting. The amount of phosphorylated protein was quantified as in **b**. Results are representative of two independent experiments. **d** A549 cells transfected with indicated siRNAs were starved (3 h) and restimulated (10 min) with amino acids (a.a.). Proteins were coimmunostained with LAMP2 (red) and mTOR (green) antibodies. Merged figures are shown in the bottom panels. Images are representative of two independent experiments. Scale bar, 10 μm. **e** A549 cells were treated with siRNAs as indicated. Electron-dense lysosome-like organelle was observed by electron microscopy. The lower graph show the quantification of the average distance between the centroid of the nucleus and the lysosome-like organelles. Images are representative of three independent experiments. Scale bars, 2 μm. **P < 0.01. **f** MKN45 cells transfected with indicated siRNAs were starved (3 h) or starved and restimulated (10 min) with amino acids (a.a.). Cell lysates were analyzed by immunoblotting. Results are representative of two independent experiments. The relative amount of phosphorylated protein was quantified as in **b**. Note that we consistently observed amino acid stimulation paradoxically reduces the phosphorylation of S6K1 but not S6 in MKN45 cells. It is possible that some differences in the composition of dialyzed serum affect the phosphorylation of S6K1. **g** MKN45 cells were starved of amino acids for 3 h, and cells were stained with antibodies as indicated. Images are representative of two independent experiments. Scale bar, 10 μm. **h** A549 cells were treated as in **d**. Proximity of mTOR to the lysosome was detected by in situ PLA using mTOR and LAMP2 antibodies. Cell nuclei were counter-stained by DAPI. Images are representative of three independent experiments. Scale bar, 10 μm.

In response to various physiological stimuli, including growth factors, energy status and amino acids, mTORC1 regulates multiple types of cellular processes, such as mRNA translation (Fig. 2a). We found that TUFT1 depletion in A549 cells inhibited insulin-induced phosphorylation of 70-kDa ribosomal S6 kinase (S6K1), a downstream effector of mTORC1 signaling, without decreasing upstream AKT phosphorylation (Fig. 2b). The results also indicated that knockdown of TUFT1 partially increased the amount of phosphorylated AKT in A549 cells, possibly by a feedback regulation^33^. S6K1 phosphorylation in response to stimulation with nutrients was also decreased in MDA-231-D cells (Supplementary Figure S3A). To determine the physiological functions of TUFT1 in vivo, we established *Tuft1* mutant mice harboring a gene trap allele, *Tuft1*^*tm1a(KOMP)Wtsi*^^34^. Surprisingly, all *Tuft1* homozygous mutant mice died within hours postnatally, indicating the importance of TUFT1 in mammalian development. We therefore generated primary MEF cells lacking *Tuft1*. Consistent with our earlier results, phosphorylation of S6K1 was lower in *Tuft1* mutant MEF cells than in wild-type MEF cells (Fig. 2c).

We then examined the effect of TUFT1 on mTORC1 recruitment to the lysosomal surface. In cells with TUFT1 knockdown, both mTOR and the lysosomes were diffusely located in the cytoplasm even after stimulation with amino acids (Fig. 2d). Electron microscopy analysis also indicated an inhibition of perinuclear accumulation of electron-dense organelles, which comprise lysosomes and other lysosome-related organelles, in TUFT1-depleted cells (Fig. 2e). Amino acids activate mTORC1 through heterodimers of the Rag subfamily of small GTPases^35^. To assess the involvement of TUFT1 in amino acid signaling, we utilized MKN45 human gastric cancer cells, in which the *NPRL3* gene is homozygously deleted, and Kato III as control human gastric cancer cells (Supplementary Figure S3B). NPRL3 is a component of the GATOR1 complex which displays GAP activity for RagA/B; mTORC1 signaling was therefore constitutively activated and mTORC1 was localized at the lysosomes in MKN45 cells irrespective of amino acid stimulation^36^. We observed consistent results concerning the activation of mTORC1 signal in the starved MKN45 cells, and knockdown of TUFT1 in MKN45 cells decreased the phosphorylation of S6K1 and S6 protein (Fig. 2f). In addition, we found that amino acid stimulation paradoxically reduced the phosphorylation of S6K1 and its target S6, through an unknown mechanism in our experimental condition in MKN45 cells. Knockdown of TUFT1 also caused diffuse localization of mTOR and the lysosomes in these cells (Fig. 2g). In contrast, amino acid stimulation increased the phosphorylation of S6K1 in Kato III cells (Supplementary Figure S3C), and the effect of amino acids was abolished in TUFT1-silenced Kato III cells. The eukaryotic translation initiation factor 4E-binding protein 1 (4E-BP1) is another well-characterized mTORC1 target. We confirmed depletion of TUFT1 inhibited phosphorylation of 4E-BP1 induced by amino acid stimulation in Kato III cells (Supplementary Figure S3C). Translocation of endogenous mTOR to the lysosomal surface was then examined by an in situ proximity ligation assay (PLA), an immunoassay that enables detection of proximity between two proteins. PLA signals between the antibodies for mTOR and LAMP2 were detected both in control cells and cells with TUFT1 knockdown (Fig. 2h), indicating close proximity between mTOR and LAMP2. These results show that TUFT1 targets mTORC1 signaling, independently of Rag signaling-induced recruitment of mTORC1 to the lysosomes.

### TUFT1 controls the network of multiple cellular compartments

Our data raised the possibility that TUFT1 regulated lysosomal positioning, which may determine the surroundings of the lysosomes and mTORC1 activity. To investigate whether lysosomal dispersion affected lysosomal functions, we analyzed the turnover rate of epidermal growth factor receptor (EGFR) after EGF stimulation. However, immunoblot analysis revealed that there was almost no change in the degradation rate of EGFR in TUFT1 siRNA-treated cells (Supplementary Figure S4A). Moreover, depletion of TUFT1 did not affect lysosomal acidification as assessed by LysoTracker staining intensity (Supplementary Figure S4B), or lysosomal aspartic protease cathepsin D processing (Supplementary Figure S4C). Collectively, these results suggested that depletion of TUFT1 changed lysosomal positioning but did not reduce lysosomal function.

**Fig. 3:**
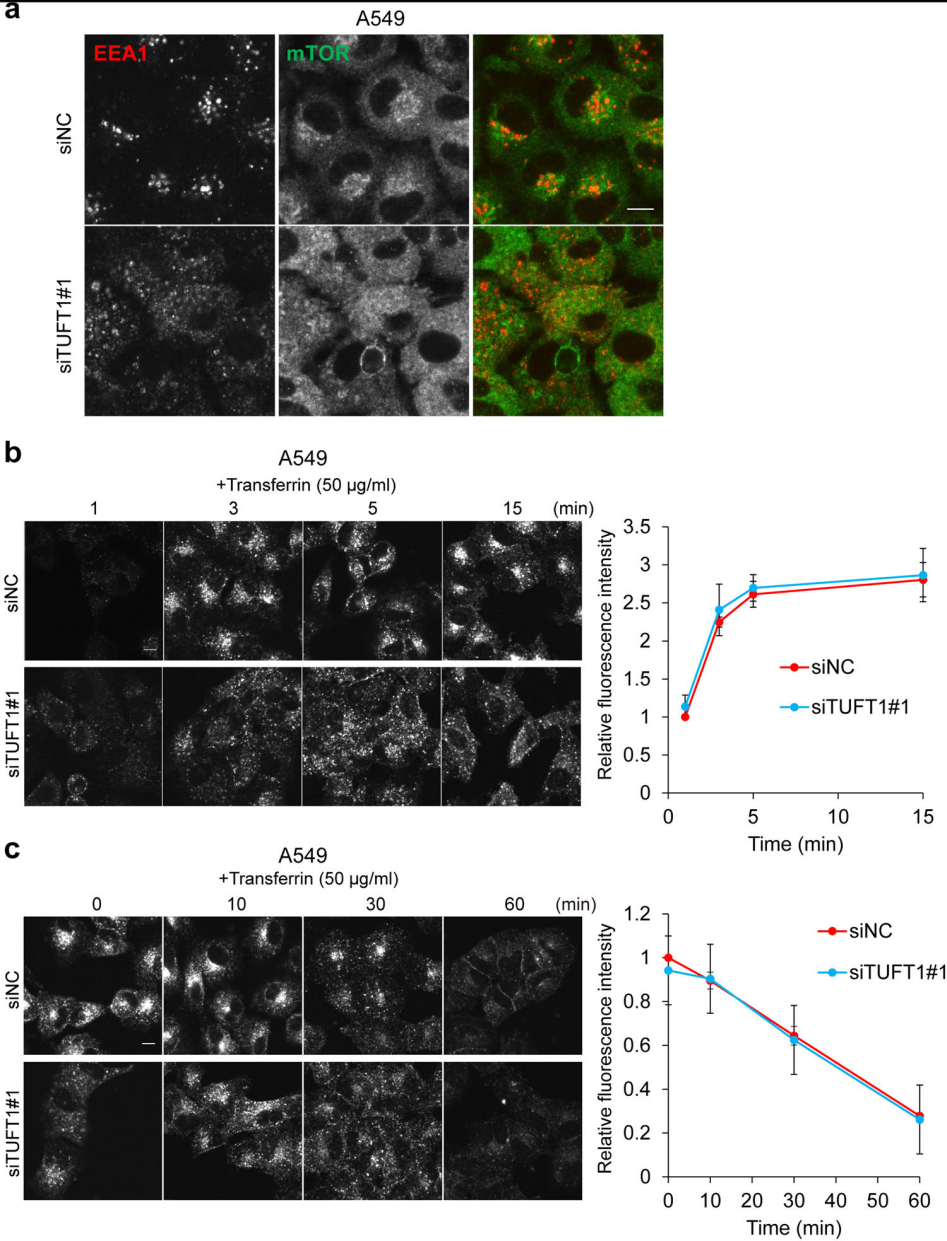
TUFT1 downregulation causes dysregulation of intracellular compartment positioning and vesicular trafficking. **a** A549 cells that were starved (3 h) and restimulated (10 min) with nutrients were coimmunostained with antibodies for EEA1 (red) and mTOR (green). Images are representative of two independent experiments. Scale bar, 10 μm. **b** A549 cells transfected with the indicated siRNAs were serum starved for 1 h and incubated with Alexa594-conjugated transferrin (50 μg/mL) at 37 °C for the indicated time periods. Representative fields are shown in the left panel. Images are representative of two independent experiments. Scale bar, 10 μm. In the right panel, the internalized transferrin fluorescence intensity is shown. Results are means ± s.d. of two independent experiments. **c** A549 cells transfected with the indicated siRNAs were serum starved for 1 h and pulsed with Alexa594-conjugated transferrin (50 μg/mL) at 37 °C for 15 min. Cells were fixed at the indicated time points. Representative fields are shown in the left panel. Images are representative of two independent experiments. Scale bar, 10 μm. In the right panel, the amount of transferrin in the cells was quantified using ImageJ software. Results are means ± s.d. of two independent experiments.

We next investigated the involvement of TUFT1 in positioning of non-lysosomal cellular compartments and vesicular trafficking. Similar to LAMP2, the early endosomal marker EEA1 was localized diffusely throughout the cytoplasm in TUFT1-deficient cells (Fig. 3a). In addition, the distribution of Alexa594-conjugated transferrin after the endocytosis was observed at more peripheral regions in TUFT1-depleted cells (Fig. 3b). We then tested for recycling defects in these cells. Although TUFT1 depletion did not affect recycling efficiency as detected by a loss of fluorescence (Fig. 3c), the subcellular components containing fluorescence-labeled transferrin were mislocalized. On the basis of these findings, we hypothesized that knockdown of TUFT1 resulted in altered positioning of cellular compartments and vesicular trafficking that affected mTORC1 signaling.

### Distribution and dynamics of cellular compartments determine mTORC1 activity

Our present findings suggested the possibility that TUFT1 regulates positioning of cellular compartments, vesicular trafficking and mTORC1 signaling by altering the actin cytoskeletal organization (Fig. 1c). We then disrupted the actin cytoskeleton with cytochalasin D (Supplementary Figure S5A) and determined its effects on lysosomal localization and mTORC1 activation. As previously reported^37^, treatment with cytochalasin D had little effect on subcellular localization of mTOR and the lysosomes (Supplementary Figure S5B). Cytochalasin D did not affect phosphorylation of S6K1, S6 or 4E-BP1 (Supplementary Figure S5C). These results suggest that TUFT1 regulates mTORC1 signaling activity in a RhoA-actin-independent manner.

**Fig. 4:**
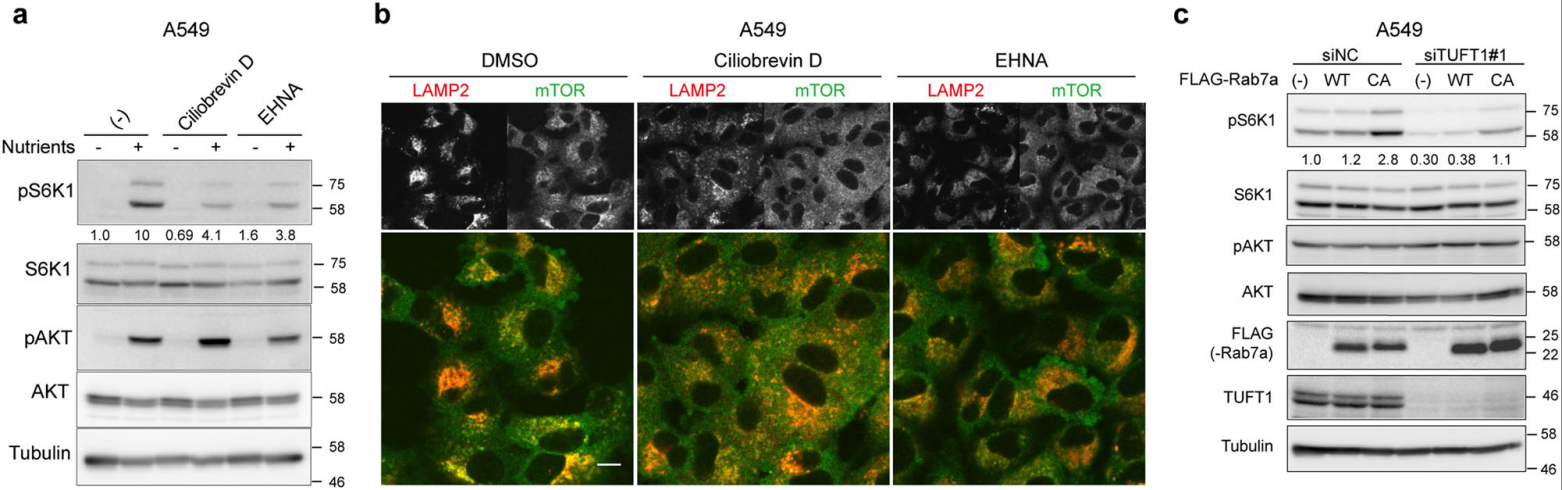
Dysregulation of vesicular trafficking leads to mTORC1 inactivation. **a** A549 cells were starved (2 h), incubated with ciliobrevin D (50 µM) or EHNA (500 µM) for 1 h, and restimulated (10 min) with nutrients. Cell lysates were collected for immunoblotting. The relative amount of phosphorylated protein was quantified as in Fig. 2b. Results are representative of two independent experiments. **b** A549 cells were treated as in **a**. Cells were immunostained with antibodies for LAMP2 (red) and mTOR (green). Merged figures are shown in the bottom panels. Images are representative of two independent experiments. Scale bar, 10 μm. **c** A549 cells transfected with indicated siRNAs and Rab7a expression plasmids were starved (3 h) and restimulated with nutrients (10 min). Cell lysates were analyzed by immunoblotting. The relative amount of phosphorylated protein was quantified as in Fig. 2b. Results are representative of two independent experiments. CA, constitutively active form.

We then manipulated retrograde transport with the cytoplasmic dynein inhibitors ciliobrevin D and erythro-9-(2-hydroxy-3-nonyl) adenine (EHNA). We found that these inhibitors caused mislocalization of early endosomes, the localization of which is regulated by dynein activity^38^ (Supplementary Figure S5D). As previously described^39^, the dynein inhibitors disrupted the phosphorylation of S6K1 after nutrient treatment without altering that of AKT (Fig. 4a). In addition, the dynein inhibitors caused dispersion of the lysosomes and mTOR, similar to TUFT1 depletion (Fig. 4b). In situ PLA assay using antibodies for mTOR and LAMP2 indicated that ciliobrevin D did not disrupt the colocalization of mTOR and the lysosomes (Supplementary Figure S5E). In MKN45 cells which have constitutively active RagA/B, these inhibitors also caused mTORC1 inactivation (Supplementary Figure S5F). In addition, overexpression of Kif5b, which is required for normal distribution of multiple organelles including lysosomes^40^, caused mislocalization of the lysosomes (Supplementary Figure S5G) and inhibited S6K1 phosphorylation (Supplementary Figure S5H). These results suggested that dynein-regulated vesicular trafficking was necessary for mTORC1 activity independently of Rag GTPases and PI3K-AKT signaling.

We next examined whether perinuclear clustering of the lysosomes affected mTORC1 signaling by utilizing Rab7a. We used Rab7a and its constitutively active (CA) mutant (Q67L), which was shown to cause a tight clustering of the lysosomes to the perinuclear region^41^. Overexpression of CA forms of Rab7a showed a weak effect on S6K1 phosphorylation (Fig. 4c) in TUFT1-depleted cells, indicating that the position of the lysosomal compartment partially affected the mTORC1 activity. The present findings also suggested that the effect of TUFT1 depletion on mTOR signaling is mediated by some other mechanisms independent of the lysosomal localization.

### TUFT1 interacts with RABGAP1, a GAP for certain Rab GTPases

**Fig. 5:**
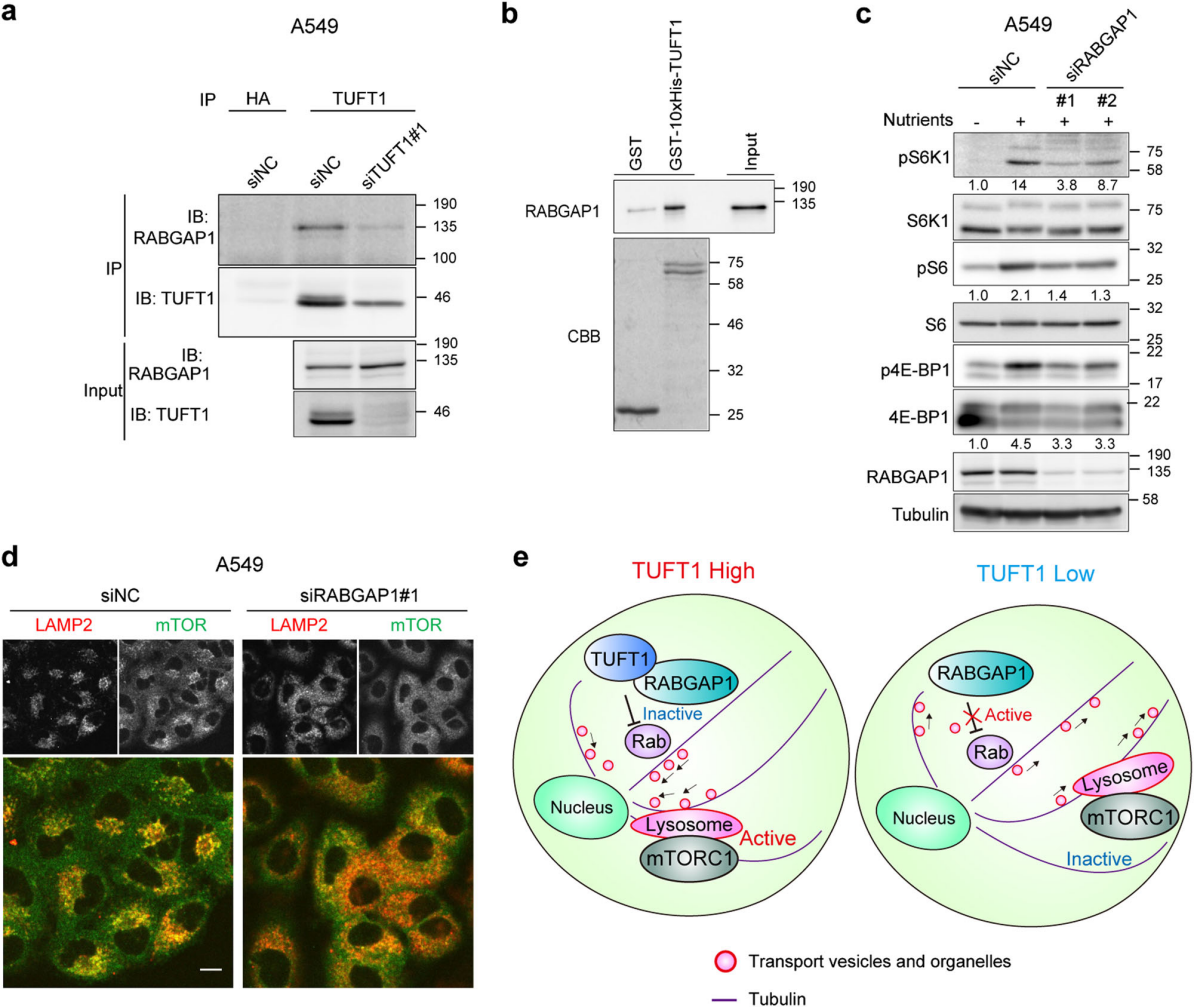
RABGAP1 interacts with TUFT1 and regulates lysosomal positioning and mTORC1 signaling. **a** Lysates of A549 cells transfected with the indicated siRNAs were subjected to immunoprecipitation using HA or TUFT1 antibodies. Immunoprecipitates and 3% input extracts were immunoblotted with the indicated antibodies. Results are representative of two independent experiments. **b** GST pull-down assays were performed with GST-10xHis-TUFT1, negative control GST, and recombinant RABGAP1. Specific interaction of these proteins was detected by immunoblotting. The lower panel demonstrates the comparable GST fusion protein loading on the lanes. CBB, Coomassie Brilliant Blue staining. **c** A549 cells transfected with the indicated siRNAs were starved (3 h) or starved and restimulated (10 min) with nutrients. Cell lysates were analyzed by immunoblotting. The relative amount of phosphorylated protein was quantified as in Fig. 2b. Results are representative of two independent experiments. **d** A549 cells transfected with the indicated siRNAs were starved (3 h) and restimulated (10 min) with nutrients, then immunostained with antibodies for LAMP2 (red) and mTOR (green). The merged images are presented in the bottom row. Images are representative of two independent experiments. Scale bar, 10 μm. **e** TUFT1 modulates lysosomal positioning, vesicular trafficking and mTORC1 signaling through its interactions with RABGAP1. A working model illustrating the role of TUFT1-RABGAP1 in lysosomal positioning, vesicular trafficking and mTORC1 activation.

By searching BioPlex, a human protein interactome database in HEK293T cells^42^, we identified an interaction between TUFT1 and RABGAP1 (also known as TBC1D11 or GAPCenA), which belongs to the group of TBC (TRE2-BUB2-CDC16) domain proteins. The physiological functions of RABGAP1 are poorly understood. We found that exogenously expressed RABGAP1 and TUFT1 co-immunoprecipitated in HEK293T cells (Supplementary Figure S6A), and endogenous TUFT1 and RABGAP1 in A549 cells also co-immunoprecipitated (Fig. 5a and Supplementary Figure S6B). In addition, to test whether TUFT1 directly interacts with RABGAP1, we performed GST pull-down assays using recombinant proteins, and found that TUFT1 specifically interacted with RABGAP1 in vitro (Fig. 5b). Compared to control A549 and MDA-231-D cells, those depleted of RABGAP1 showed reduced mTORC1 signaling (Fig. 5c and Supplementary Figure S6C). Moreover, the lysosomes (Fig. 5d) and early endosomes (Supplementary Figure S6D) were dispersed in cells transfected with RABGAP1 siRNAs. In addition, PLAs using mTOR-LAMP2 antibodies demonstrated that depletion of RABGAP1 did not disrupt nutrient-mediated mTORC1 translocation to the lysosomes (Supplementary Figure S6E).

Rab GAPs inactivate their target Rab GTPases by converting them from the GTP-bound to the GDP-bound states. To determine whether the effect of RABGAP1 is GAP activity dependent, we established A549 cells stably expressing either control (GFP), wild-type RABGAP1 (RABGAP1-WT) or GAP activity-deficient RABGAP1 mutant (R612A)^43^, and performed rescue experiments (Supplementary Figure S6F). The result showed that only RABGAP1-WT could rescue the knockdown phenotype, indicating that GAP activity of RABGAP1 is required for mTORC1 activity.

RABGAP1 stimulates the GTPase activity of several Rab proteins, including Rab4, Rab6, Rab11 and Rab36^44–46^. Among them, we confirmed that wild-type and CA forms (Q182L) of Rab36, but not the dominant negative (DN) form (T137N), interacted with RABGAP1 (Supplementary Figure S6G). In addition, overexpression of the CA-form of Rab36 partially suppressed the phosphorylation of S6K1 (Supplementary Figure S6H) in HEK293T cells, suggesting that active Rab36 regulated mTORC1 signaling. Effect of TUFT1 on RABGAP1 activity was also evaluated. Although our findings suggested that the substrate of RABGAP1 is not limited to Rab36 for regulation of mTORC1 activity, the result suggested that TUFT1 up-regulated GAP activity of RABGAP1 (Supplementary Figure S6I). TUFT1 therefore coordinates with RABGAP1 to control the dynamics of vesicular trafficking and mTORC1 signaling by regulating some Rab GTPases including Rab36 or some other molecules (Fig. 5e).

### TUFT1 expression is useful for estimating sensitivity of perifosine

**Fig. 6:**
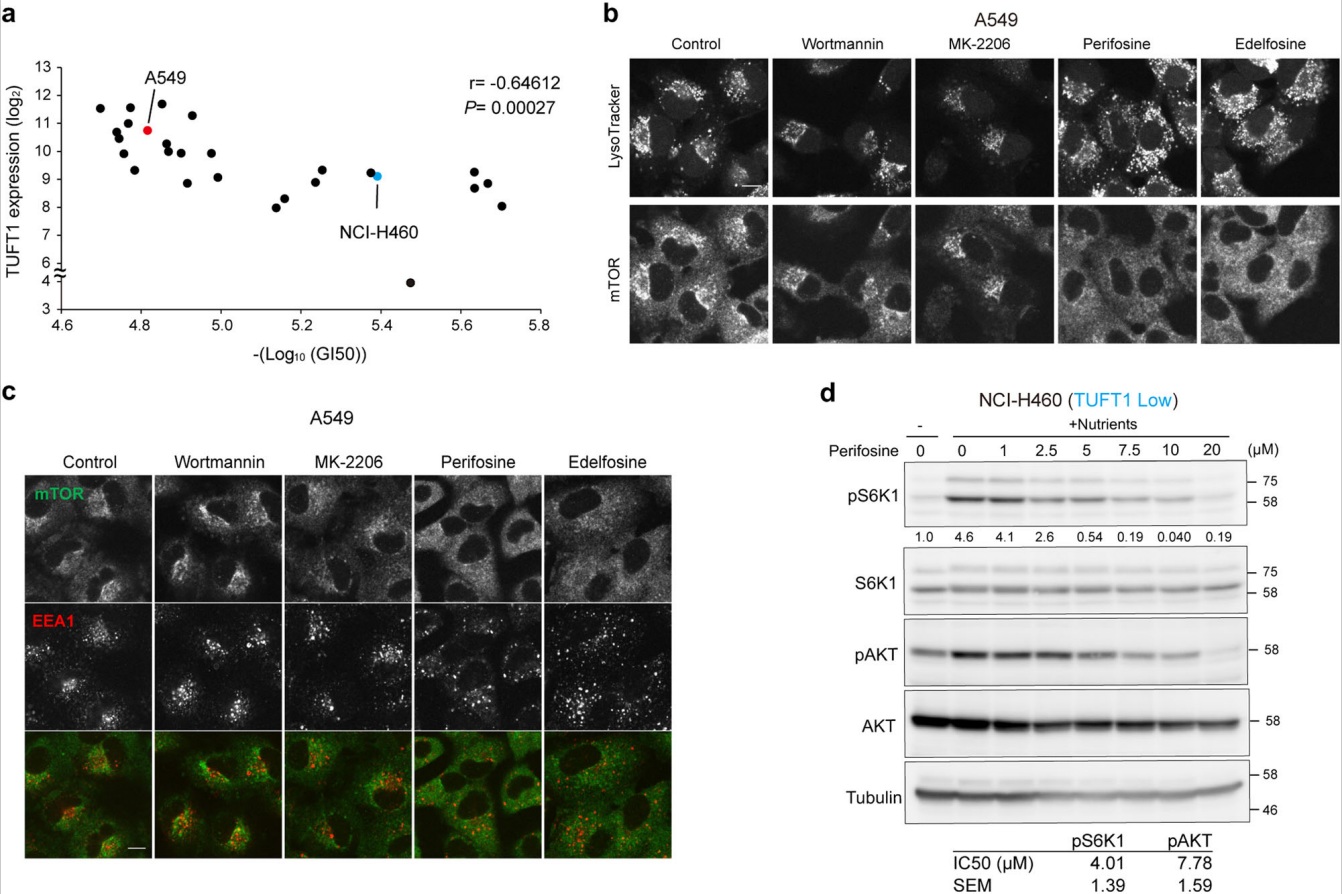
Perifosine targets vesicular trafficking and mTORC1 signaling. **a** Relationship between *TUFT1* expression and perifosine sensitivity. The Pearson correlation coefficient (r) and p-value (*P*) are shown. Twenty-seven cells, of which we obtained both *TUFT1* expression profiles from CCLE and 50% growth inhibition (GI50) values of perifosine, were analyzed. **b** A549 cells were treated with wortmannin (2 μM), MK-2206 (2 μM), perifosine (20 μM) or edelfosine (10 μM). Cells were starved (2 h) in the presence of the indicated inhibitors and with LysoTracker Red (DND-99) for an additional 1 h. Cells were then restimulated with nutrients (10 min). Proteins were immunostained with antibody for mTOR. Images are representative of three independent experiments. Scale bar, 10 μm. **c** A549 cells were starved (3 h) in the presence of the indicated inhibitors and restimulated (10 min) with nutrients. Cells were fixed and immunostained with EEA1 (red) and mTOR (green) antibodies. Merged figures are shown. Scale bar, 10 μm. Images are representative of two independent experiments. **d** NCI-H460 cells were starved (3 h) or starved and restimulated (10 min) with nutrients in the absence or presence of perifosine at indicated concentrations. Cell lysates were subjected to immunoblot analysis. The relative amount of phosphorylated protein was quantified as in Fig. 2b. Results are representative of three independent experiments. Calculated IC50 values are shown in the bottom table.

To address the potential of TUFT1 expression and related intracellular compartment positioning and vesicular trafficking as a molecular target of cancer treatment, we examined correlations between drug action and *TUFT1* expression obtained from the data provided by Cancer Cell Line Encyclopedia (CCLE)^47^ and our published database of drug sensitivity across a panel of 39 human cancer cell lines (JFCR39)^48, 49^. We performed correlation analysis using conventional anti-cancer drugs as well as PI3K-AKT-mTOR pathway inhibitors. We found a significant sensitivity to perifosine, which negatively correlated with *TUFT1* expression in the cell line panel (Fig. 6a and Supplementary information, Table S1). Perifosine, an alkylphospholipid PI3K-AKT inhibitor, is an anti-tumor compound that alters the composition of lipid rafts of the plasma membrane, and has been subjected to phase III trials for colorectal cancer and multiple myeloma. However, the mechanism of action of perifosine has not been fully elucidated due to its wide range of actions.

We then evaluated the effect of perifosine on lysosomal positioning, one of the points of action of TUFT1. A549 cells were treated with perifosine, its analog edelfosine, the PI3K inhibitor wortmannin or the AKT inhibitor MK-2206; these drugs inhibited AKT and S6K1 phosphorylation (Supplementary Figure S7A). Under these conditions, perifosine and edelfosine totally disrupted mTOR and lysosomal accumulation, while wortmannin or MK-2206 failed to do so (Fig. 6b). Perifosine and edelfosine also caused early endosomal dispersion (Fig. 6c), indicating that perifosine targeted the vesicular trafficking system. Next, we evaluated the effects of TUFT1 overexpression on the localization of the lysosomes, and found that overexpression of TUFT1 reduced the sensitivity of A549 cells to perifosine (Supplementary Figure S7B). In addition, we compared the sensitivity of A549 (high expression of *TUFT1*) and NCI-H460 (low expression of *TUFT1*) cancer cells to perifosine in terms of the distribution of mTORC1 and lysosomes. Calculated GI50 (50% growth inhibition) of perifosine was 4.06 μM (NCI-H460) and 15.3 µM (A549) (Fig. 6a). Consistent with the above findings, A549 cells were more resistant to perifosine in terms of mTORC1 and lysosomal translocation than NCI-H460 cells (Supplementary Figure S7C and D). Although perifosine inhibited the phosphorylation of both AKT and S6K1, perifosine inhibited phosphorylation of S6K1 at lower concentrations than that of AKT in NCI-H460 cells (Fig. 6d). These results indicated the utility of perifosine as an inhibitor of mTORC1 signaling, which inhibited both AKT and mTOR activities.

## Discussion

The present study identified TUFT1 as a factor associated with poor prognosis in several types of cancers. We also showed that this protein regulates tumor growth and metastasis in vitro and in vivo. Our results here showed that TUFT1, which likely acts as an adaptor or an effector for RABGAP1, regulated lysosomal positioning and vesicular trafficking. The vesicular trafficking network is extensively integrated into various signaling pathways. Among them, the results of RNA sequencing and other in vitro experiments indicated that TUFT1 is involved in mTORC1 signaling.

Normally controlled positioning of intracellular compartments and vesicular trafficking have been suggested to be required for mTORC1 activation. RNAi screening showed that knockdown of several Rab GTPases decreases phosphorylation of *Drosophila* S6K in *Drosophila* S2 cells^17^. Overexpression of Rab5 inhibits mTORC1 signaling and mislocalization of mTOR to enlarged, swollen vacuolar structures^50^. Knockdown of Rab12 activates mTORC1 signaling by affecting cell surface abundance of the amino acid transporter PAT4^51^. Consistently, disruption of the vesicular trafficking system by dynein inhibitors inhibited mTORC1 activation, suggesting that cycling of intact Rab GTPases and their cellular trafficking are required for mTORC1 activation.

Dynein-dependent and Rab-dependent intracellular compartment positioning could play an important role in mTORC1 signaling. The relationship between lysosomal positioning in cells and mTORC1 activity is controversial; lysosomal positioning to the cell periphery has been reported to enhance mTORC1 activity in HeLa cells^15^, whereas perinuclear accumulation of lysosomes and mTORC1 hyper-activation were observed in a cellular model of Huntington’s disease^16^. We here showed that the disturbance of the cytoskeletal motor activity by overexpression of Kif5b, which reduced the perinuclear accumulation of the lysosomes, caused inactivation of mTORC1 in A549 cells^40^. These results appear to support the importance of perinuclear accumulation of lysosomes for mTORC1 activity. However, overexpression of Rab7a-CA, which triggered the accumulation of lysosomes to the perinuclear region^41^, could only partially rescue the knockdown effect of TUFT1. The present findings therefore suggest that TUFT1-RABGAP1 regulates mTORC1 signaling by maintaining normal vesicular trafficking, possibly in part by accumulating the lysosomes to perinuclear region, although some other mechanisms may be involved in this phenomenon.

We identified RABGAP1, which is a GAP for Rab GTPases, as a binding partner for TUFT1. It was also confirmed that GAP activity of RABGAP1 is required for mTORC1 activity. RABGAP1 reportedly targets Rab4, Rab6, Rab11, and Rab36^44, 45^. Among them, Rab36 has been identified as a candidate target of RABGAP1 in the present study. In addition, the presence of TUFT1 could strengthen the GAP activity of RABGAP1 on Rab36. Although Rab36 does not appear to be the only target of RABGAP1-TUFT1 for the regulation of mTORC1 signaling, these results regarding Rab36 provide important insights into the mechanism of TUFT1 function through RABGAP1 and target Rab GTPases, including Rab36.

Vesicular trafficking is involved in various cellular functions, such as cell differentiation, transformation, cytoskeleton formation, and invasion^52^. The expression of some Rab GTPase-encoding genes are changed in several human cancers^53^. Dysfunction of vesicular trafficking is thus considered to be involved in cancer progression, but its mechanisms remain unclear. We showed here that depletion of TUFT1 or RABGAP1 alleviated the accumulation of transport vesicles and lysosomes, which led to mTORC1 inactivation and tumor regression in A549 lung adenocarcinoma cells. In addition, treatment with dynein inhibitors also suppressed mTORC1 activity, indicating that these procedures could recover normal vesicular trafficking patterns in cancer cells. Regulation of mTORC1 signaling by TUFT1-RABGAP1 may thus be a factor that links vesicular trafficking and tumor progression. These findings suggest that regulation of trafficking system may be a promising target for cancer therapy.

The PI3K-AKT-mTOR signaling pathway is frequently activated in cancers^54^, suggesting mTOR as a promising target for cancer therapy. However, drugs that target the PI3K-AKT-mTOR signaling pathway have exhibited limited efficacy. Our drug sensitivity test indicated that the effect of perifosine, an alkylphospholipid PI3K-AKT inhibitor, correlated with *TUFT1* expression. Unlike other AKT inhibitors, perifosine directly binds the PH domain of AKT, preventing its translocation to lipid rafts of the plasma membrane, which serve as scaffolds for important signal transduction components^55^. In addition, our results suggested a mechanism of action of perifosine that is related to TUFT1. Perifosine inhibited mTORC1 activation by affecting lysosomal positioning and subcellular vesicular trafficking. In addition, TUFT1-mediated vesicular trafficking may be involved in the mechanisms in the generation of drug resistance. Zhou and colleagues described a role of TUFT1 in the metastasis of pancreatic cancer cells by increasing hypoxia-inducible factor-1-Snail signaling which promotes EMT^22^. We did not detect changes in EMT markers under our experimental conditions; however, TUFT1 may have different functions in some contexts through the regulation of vesicular trafficking. Thus, further research may position TUFT1 as a biomarker or as a therapeutic target for various cancers.

## Materials and methods

### Cell culture

A549 cells were from Cell Resource Center for Biomedical Research, Institute of Development, Aging, and Cancer, Tohoku University. A549, HEK293T, MDA-231-D (a highly metastatic variant of MDA-MB-231)^29^, and MEF cells were cultured in Dulbecco’s Modified Eagle’s Medium (DMEM #11965; Thermo Fisher Scientific) supplemented with 10% fetal bovine serum (FBS #SH30910.03; Thermo Fisher Scientific), 100 U/mL penicillin G, and 100 µg/mL streptomycin. NCI-H441 and MKN45 cells were maintained in Roswell Park Memorial Institute medium 1640 (RPMI 1640 #11875; Thermo Fisher Scientific) supplemented with 10% FBS, 100 U/mL penicillin G, and 100 µg/mL streptomycin. NCI-H460-Luc cells were obtained from JCRB Cell Bank and maintained in RPMI 1640 supplemented with 15% FBS, 0.023 IU/mL insulin, 100 U/mL penicillin G, and 100 µg/mL streptomycin. Cells were grown in a humidified atmosphere with 5% CO_2_ at 37 °C. Mycoplasma contamination was routinely checked by e-Myco VALiD Mycoplasma PCR detection kit (CosmoBio).

### Reagents and antibodies

Recombinant TGF-β (TGF-β3) was purchased from R&D Systems. Fluorescein Isothiocyanate Labeled Phalloidin (#P5282), LysoTracker Red (DND-99) (#L7528), Alexa-488 conjugated transferrin (#T13342), perifosine (#SML0612), and edelfosine (#SML0332) were obtained from Sigma-Aldrich. MK-2206 (#ENZ-CHM164) was from Enzo Life Sciences. Cytochalasin D (#037–17561) was from Wako Pure Chemical Industries. Ciliobrevin D (#250401) was obtained from Merck Millipore. EHNA (#13352) was purchased from Cayman Chemical.

Antibodies against the following proteins were purchased from Cell Signaling Technology: mTOR (#2983), pS6K1 (Thr389) (#9234), S6K1 (#9202), pAKT (Thr308) (#2965), AKT (#9272), pS6 (Ser240/244) (#2215), S6 (#2217), p4E-BP1 (Ser65) (#9451), 4E-BP1 (#9644) and pERK1 and 2 (pERK1/2) (Thr202/Tyr204) (#9101). Antibodies against TUFT1 (#sc-47536), cyclin D1 (#sc-718), cyclin D3 (#sc-182), RhoA (#sc-418) and cathepsin D (#sc-6487) were obtained from Santa Cruz Biotechnology. Antibodies against LAMP2 (#ab25631) and RABGAP1 (#ab153992) were purchased from Abcam. HDAC1 (#2E10), ERK1 and 2 (#05–1152) and EGFR (#05–101) antibodies were obtained from Merck Millipore. Antibody for EEA1 (#610457) was purchased from BD Biosciences. Antibody for c-Myc (#017–21871) was obtained from Wako Pure Chemical Industries. Antibodies against FLAG (M2; #F3165 and #F1804) and α-tubulin (#T6199) were obtained from Sigma-Aldrich. Antibody for Cdc42 (#ACD03) was obtained from Cytoskeleton.

### Plasmids

Plasmids encoding human TUFT1, RABGAP1, Rab36, and dog Rab7a were constructed by PCR amplification. The fragments were subcloned into pcDEF3-FLAG vector (TUFT1), pGEX-6P-1 vector (TUFT1), pcDNA3-FLAG vector (Kif5b and Rab7a), or pcDNA3-Myc vector (Rab36). Mutant Rab7a, RABGAP1 and Rab36 were generated by PCR mutagenesis. All cDNAs constructed were verified by sequencing. Plasmids encoding mouse Kif5b (pKin1B, Addgene plasmid #31604) was a gift from Dr. Anthony Brown^56^.

### Amino acid starvation and stimulation of cells

Cells were rinsed twice with DMEM with sodium pyruvate, without amino acids (#048–33575; Wako Pure Chemical Industries) containing 10% dialyzed serum (#SH30079; GE Healthcare) and incubated for 3 h. Cells were then stimulated with DMEM (#11965) containing amino acids and 10% FBS as indicated.

### Nutrient starvation and stimulation of cells

Cells were rinsed twice with amino acids (Arg, Leu, and Lys)-free DMEM (#D9443; Sigma-Aldrich) and incubated for 3 h. Cells were then stimulated with DMEM (#11965) containing amino acids and 10% FBS as indicated.

### Knockout mouse production

All animal experiments were performed in accordance with policies of the Animal Ethics Committee of the University of Tokyo. ES cells harboring *Tuft1*^*tm1a(KOMP)Wtsi*^ allele (EPD0383_4_G08) were obtained from the International Mouse Phenotype Consortium (http://www.mousephenotype.org) and cultured in the medium containing 3i components^57^. C57BL/6N female mice and ICR mouse strains were used as embryo donors and foster mothers, respectively. Animals were genotyped using DNA extracted from tail segments. Wild-type and mutant alleles were detected by multiplex PCR with the same reverse primer (WT-R: 5′-CCCTGAGGGACCAGCCACATAGAACAGA-3′) and different forward primers (mutant allele, Tuft1-F: 5′-TGGTCTGAGCTCGCCATCAGTTTCA-3′; wild-type allele, WT-F: 5′-CTGTTAGCATTCTGTCTAAACTTCACCCCA-3′).

### Subcutaneous xenograft model

BALB/c *nu/nu* mice (4 weeks of age) were obtained from Sankyo Labo Service. Cells (5 × 10^5^) were injected into each mouse. Subcutaneous tumors were measured externally, and tumor volume was calculated as previously described^58^.

### In vivo bone metastasis model

Stably transfected MDA-231-D-luc cells (1 × 10^5^ cells), which harbored luciferase, were injected into the left ventricle of female nude mice. Five weeks after injection, bone metastatic cells were analyzed by bioluminescence imaging.

### Electron microscopy

A549 cells were transfected with siRNA for TUFT1 or a negative control siRNA (siNC). For electron microscopic analysis, cells were treated as previously described^59^.

### Lentivirus production and infection

Lentiviral shRNA expression vectors were generated as described^29^. We used the following oligonucleotides (5′→3′): Human TUFT1 #1, GATCCCCGCTGGTCATTCTCTGGCTTACGTGTGCTGTCCGTAAGCCAGAGAATGACCAGCTTTTTGGAAAT and CTAGATTTCCAAAAAGCTGGTCATTCTCTGGCTTACGGACAGCACACGTAAGCCAGAGAATGACCAGCGGG; Human TUFT1 #2: GATCCCCGGATATAAGTAGCAAGCTTACGTGTGCTGTCCGTAAGCTTGCTACTTATATCCTTTTTGGAAAT and CTAGATTTCCAAAAAGGATATAAGTAGCAAGCTTACGGACAGCACACGTAAGCTTGCTACTTATATCCGGG.

Lentiviral expression vectors were obtained from Dr. Hiroyuki Miyoshi (RIKEN BioResource Center. current address: Keio University, Tokyo, Japan). shRNAs were transferred into the lentivirus vector CS-RfA-EG through the pENTER-4H1 vector. To produce lentivirus, HEK293FT cells were transfected with the vector constructs pCMV-VSV-G-RSV-Rev and pCAG-HIVgp. Virus was collected, concentrated with the Lenti-X Concentrator (Takara Bio), and used to infect A549 cells and MDA-231-D cells.

### RNAi in mammalian cells

RNAi was carried out with the following siRNAs designed from siDirect (RNAi inc.) (5′→3′): Human TUFT1#1, GGAUAUAAGUAGCAAGCUUGA and AAGCUUGCUACUUAUAUCCUC; Human TUFT1#2, GUAGCCUUUUGCGGAAAAAUU and UUUUUCCGCAAAAGGCUACUC; Human mTOR#1, GAUCUCAUGGGCUUCGGAACA and UUCCGAAGCCCAUGAGAUCUU; Human mTOR#2, CCAAUUAUACCCGUUCUUUAG and AAAGAACGGGUAUAAUUGGUU; Human LAMP2#1, GAUAAGGUUGCUUCAGUUAUU and UAACUGAAGCAACCUUAUCCU; Human LAMP2#2, GCUCUACUUAGACUCAAUAGC and UAUUGAGUCUAAGUAGAGCAG; Human RABGAP1#1, GGGAUAUUAACCGAACAUUCC and AAUGUUCGGUUAAUAUCCCGG; Human RABGAP1#2, GACGCAUGUUGGUAGGUCACU and UGACCUACCAACAUGCGUCUA. For human SMAD4 siRNA, Stealth Select siRNA (HSS106256, UAAGGCACCUGACCCAAACAUCACC) was used (Thermo Fisher Scientific), with Negative Control Med GC Duplex #2 (12935112, Thermo Fisher Scientific, sequence not available) as a control.

siRNAs were transfected into cells using Lipofectamine RNAiMAX reagent (Thermo Fisher Scientific) in accordance with the manufacturer’s instructions. The final concentration of siRNA in the culture medium was 10 nM.

### Transfection of cDNA

Transient transfection into cells was performed using Lipofectamine 2000 or Lipofectamine 3000 reagent (Thermo Fisher Scientific), as recommended by the manufacturer’s protocol.

### Adenovirus production and infection

Ad-LacZ and Ad-TUFT1 were prepared using pAd/CMV/V5-DEST vector. The crude adenoviral lysate was purified using ViraKit (VIRAPUR). Titration was performed by the Adeno-X Rapid Titer Kit (Clontech).

### Immunoblotting

All experiments were done at least twice with similar results. Cultured cells were rinsed with ice-cold phosphate-buffered saline (PBS) and lysed with lysis buffer (1% NP-40, 150 mM NaCl, 20 mM Tris-HCl (pH 7.5), and cOmplete EDTA-free protease inhibitor (Roche)). After centrifugation at 15,000 rpm at 4 °C for 10 min, protein concentrations were estimated with the BCA Protein Assay Kit (Thermo Fisher Scientific). Total cell lysates were subjected to sodium dodecyl sulfate polyacrylamide gel electrophoresis and transferred to FluoroTrans W membranes (Pall). Immunoblotting was carried out using the indicated antibodies, and imaging was performed with a LAS-4000 lumino-image analyzer (FUJIFILM). Band intensity was measured using ImageJ 1.50b (National Institutes of Health).

### Immunoprecipitation

Cultured cells were lysed as described above. Immunoprecipitation was performed as previously described^60^.

### GST pull-down assay

GST fusion protein of TUFT1 and control GST were prepared in *Escherichia* *coli*. Recombinant RABGAP1 was obtained from Abcam (#ab161730). The RIPA buffer (50 mM Tris-HCl (pH 8.0), 150 mM NaCl, 0.1% SDS, 0.5% DOC, 1% Triton X-100) was used for the binding assay between TUFT1 and RABGAP1.

### Nuclear/cytoplasmic fractionation

Nuclear/cytoplasmic fractionation of A549 cells was performed using the NE-PER Nuclear and Cytoplasmic Extraction Kit (Thermo Fisher Scientific) in accordance with the manufacturer’s instructions.

### Immunofluorescence microscopy

Cells were seeded in eight-well chamber slides (Thermo Fisher Scientific) and cultured or stimulated as described. Cells were fixed for 15 min in 4% paraformaldehyde in PBS, permeabilized in PBS containing 0.1% Triton X-100 for 7 min, and blocked in Blocking One (Nacalai Tesque) for 1 h at room temperature. After rinsing in PBS, slides were incubated with primary antibodies in Blocking One for 12–16 h at 4 °C and washed in PBS. Secondary antibodies were diluted in Blocking One and incubated with the slides for 1 h at room temperature. Chamber slides were mounted on glass coverslips using VECTASHIELD Antifade Mounting Medium with DAPI (Vector Laboratories). Images were obtained with an FV10i confocal laser-scanning microscope (Olympus).

### In situ PLA

Cells were cultured, fixed, and permeabilized as described above. We used the Duolink (Sigma-Aldrich) kit in accordance with the manufacturer’s protocol. Fluorescence images were obtained with an FV10i microscope. Images were collected from single focal planes.

### RNA extraction and qRT–PCR

Quantitative reverese transcriptase–PCR (qRT–PCR) was previously described^61^. For mRNA detection, total RNA was extracted with TriPure Isolation Reagent (Roche) or with the RNeasy Mini Kit (QIAGEN). First-strand cDNAs were synthesized using PrimeScript II reverse transcriptase (Takara Bio). qRT-PCR was performed with the StepOnePlus Real-Time PCR System (Thermo Fisher Scientific). All samples were run in duplicate, and results were averaged and normalized to the expression of GAPDH (which encodes glyceraldehyde-3-phosphate dehydrogenase).

### Transferrin uptake assay

Cells were starved in serum-free DMEM for 1 h. Then, cells were incubated with Alexa-488 conjugate transferrin (50 μg/mL) for the indicated periods at 37 °C. To study transferrin endocytosis and recycling, cells were incubated with Alexa-594 transferrin (50 μg/mL), washed with PBS three times, and then incubated at 37 °C for various lengths of time. Cells were fixed immediately. Images were taken with an FV10i confocal laser-scanning microscope.

### Chamber migration assay

The migration assay was performed as described previously^29^. Migrated cells were counted as field images that were selected randomly. The average number of cells was calculated from these images.

### Statistical analyses

Comparisons between two samples were performed with the Welch's t-test for in vitro data, and the Mann-Whitney-Wilcoxon test (Mann-Whitney U test) for in vivo data. Comparisons between the multiple experimental groups were made using Turkey-Kramer test for in vitro data, and Steel-Dwass test for in vivo data. Statistical analyses were conducted with the R Project for Statistical Computing (version R-3.3.2). We calculated the degree of similarity between drug sensitivity and gene expression using the Pearson correlation coefficient, as described^49^.

### RNA sequencing

Total RNA was extracted with the RNeasy Mini Kit (QIAGEN) from siRNA-transfected A549 cells. mRNA was purified using the Dynabeads mRNA DIRECT Purification Kit (Thermo Fisher Scientific). Libraries were prepared using the Ion Total RNA-Seq Kit v2 according to the manufacturer’s protocol and directionally sequenced with the Ion Proton using the Ion PI chip v2 and Ion PI IC 200 Kit (Thermo Fisher Scientific). Reads were aligned against the human genome (hg19) using TopHat2. Differential expression was evaluated using the Cuffdiff function of Cufflinks. Raw sequencing data are available at GEO (GSE99149).

## Electronic supplementary material

Supplementary Information

## References

[1] Stenmark H (2009). Rab GTPases as coordinators of vesicle traffic. Nat. Rev. Mol. Cell. Biol..

[2] Zhen Y, Stenmark H (2015). Cellular functions of Rab GTPases at a glance. J. Cell Sci..

[3] Yang PS (2011). Rab5A is associated with axillary lymph node metastasis in breast cancer patients. Cancer Sci..

[4] Nakano T (2012). Establishment of a human lung cancer cell line with high metastatic potential to multiple organs: gene expression associated with metastatic potential in human lung cancer. Oncol. Rep..

[5] Cheng KW (2004). The RAB25 small GTPase determines aggressiveness of ovarian and breast cancers. Nat. Med..

[6] Zoncu R, Efeyan A, Sabatini DM (2011). mTOR: from growth signal integration to cancer, diabetes and ageing. Nat. Rev. Mol. Cell. Biol..

[7] Jewell JL (2015). Metabolism. Differential regulation of mTORC1 by leucine and glutamine. Science.

[8] Wullschleger S, Loewith R, Oppliger W, Hall MN (2005). Molecular organization of target of rapamycin complex 2. J. Biol. Chem..

[9] Sabatini DM (2006). mTOR and cancer: insights into a complex relationship. Nat. Rev. Cancer.

[10] Yang Q, Guan KL (2007). Expanding mTOR signaling. Cell Res..

[11] Garami A (2003). Insulin activation of Rheb, a mediator of mTOR/S6K/4E-BP signaling, is inhibited by TSC1 and 2. Mol. Cell.

[12] Powis K, De Virgilio C (2016). Conserved regulators of Rag GTPases orchestrate amino acid-dependent TORC1 signaling. Cell Discov..

[13] Shimobayashi M, Hall MN (2016). Multiple amino acid sensing inputs to mTORC1. Cell Res..

[14] Laplante M, Sabatini DM (2012). mTOR signaling in growth control and disease. Cell.

[15] Erie C, Sacino M, Houle L, Lu ML, Wei J (2015). Altered lysosomal positioning affects lysosomal functions in a cellular model of Huntington’s disease. Eur. J. Neurosci..

[16] Korolchuk VI (2011). Lysosomal positioning coordinates cellular nutrient responses. Nat. Cell. Biol..

[17] Li L (2010). Regulation of mTORC1 by the Rab and Arf GTPases. J. Biol. Chem..

[18] Deutsch D (1994). Mapping of the human tuftelin (TUFT1) gene to chromosome 1 by fluorescence in situ hybridization. Mamm. Genome.

[19] Deutsch D (1991). Sequencing of bovine enamelin (“tuftelin”) a novel acidic enamel protein. J. Biol. Chem..

[20] Deutsch D (2002). The human tuftelin gene and the expression of tuftelin in mineralizing and nonmineralizing tissues. Connect. Tissue Res..

[21] Leiser Y (2007). Localization, quantification, and characterization of tuftelin in soft tissues. Anat. Rec. (Hoboken)..

[22] Zhou B (2016). TUFT1 regulates metastasis of pancreatic cancer through HIF1-Snail pathway induced epithelial-mesenchymal transition. Cancer Lett..

[23] Leiser Y (2011). The induction of tuftelin expression in PC12 cell line during hypoxia and NGF-induced differentiation. J. Cell Physiol..

[24] Saad RS, Liu YL, Han H, Landreneau RJ, Silverman JF (2004). Prognostic significance of thyroid transcription factor-1 expression in both early-stage conventional adenocarcinoma and bronchioloalveolar carcinoma of the lung. Hum. Pathol..

[25] Isogaya K (2014). A Smad3 and TTF-1/NKX2-1 complex regulates Smad4-independent gene expression. Cell Res..

[26] Ringnér M, Fredlund E, Häkkinen J, Borg Aring, Staaf J (2011). GOBO: gene expression-based outcome for breast cancer online. PLoS ONE.

[27] Gyorffy B, Surowiak P, Budczies J, Lanczky A (2013). Online survival analysis software to assess the prognostic value of biomarkers using transcriptomic data in non-small-cell lung cancer. PLoS ONE.

[28] Saito RA (2009). Thyroid transcription factor-1 inhibits transforming growth factor-beta-mediated epithelial-to-mesenchymal transition in lung adenocarcinoma cells. Cancer Res..

[29] Ehata S (2007). Ki26894, a novel transforming growth factor-beta type I receptor kinase inhibitor, inhibits in vitro invasion and in vivo bone metastasis of a human breast cancer cell line. Cancer Sci..

[30] Nobes CD, Hall A (1995). Rho, rac, and cdc42 GTPases regulate the assembly of multimolecular focal complexes associated with actin stress fibers, lamellipodia, and filopodia. Cell.

[31] Gera JF (2004). AKT activity determines sensitivity to mammalian target of rapamycin (mTOR) inhibitors by regulating cyclin D1 and c-myc expression. J. Biol. Chem..

[32] Liu L (2010). Rapamycin inhibits cytoskeleton reorganization and cell motility by suppressing RhoA expression and activity. J. Biol. Chem..

[33] Breuleux M (2009). Increased AKT S473 phosphorylation after mTORC1 inhibition is rictor dependent and does not predict tumor cell response to PI3K/mTOR inhibition. Mol. Cancer Ther..

[34] Skarnes WC (2011). A conditional knockout resource for the genome-wide study of mouse gene function. Nature.

[35] Sancak Y (2008). The Rag GTPases bind raptor and mediate amino acid signaling to mTORC1. Science.

[36] Bar-Peled L (2013). A Tumor suppressor complex with GAP activity for the Rag GTPases that signal amino acid sufficiency to mTORC1. Science.

[37] Saci A, Cantley LC, Carpenter CL (2011). Rac1 regulates the activity of mTORC1 and mTORC2 and controls cellular size. Mol. Cell.

[38] Valetti C (1999). Role of dynactin in endocytic traffic: effects of dynamitin overexpression and colocalization with CLIP-170. Mol. Biol. Cell.

[39] Clippinger AJ, Alwine JC (2012). Dynein mediates the localization and activation of mTOR in normal and human cytomegalovirus-infected cells. Genes Dev..

[40] Tanaka Y (1998). Targeted disruption of mouse conventional kinesin heavy chain, kif5B, results in abnormal perinuclear clustering of mitochondria. Cell.

[41] Taub N, Teis D, Ebner HL, Hess MW, Huber LA (2007). Late endosomal traffic of the epidermal growth factor receptor ensures spatial and temporal fidelity of mitogen-activated protein kinase signaling. Mol. Biol. Cell.

[42] Huttlin EL (2015). The BioPlex network: A systematic exploration of the human interactome. Cell.

[43] Pan X, Eathiraj S, Munson M, Lambright DG (2006). TBC-domain GAPs for Rab GTPases accelerate GTP hydrolysis by a dual-finger mechanism. Nature.

[44] Fuchs E (2007). Specific Rab GTPase-activating proteins define the Shiga toxin and epidermal growth factor uptake pathways. J. Cell Biol..

[45] Cuif MH (1999). Characterization of GAPCenA, a GTPase activating protein for Rab6, part of which associates with the centrosome. EMBO J..

[46] Kanno E (2010). Comprehensive screening for novel rab-binding proteins by GST pull-down assay using 60 different mammalian Rabs. Traffic.

[47] Barretina J (2012). The Cancer Cell Line Encyclopedia enables predictive modelling of anticancer drug sensitivity. Nature.

[48] Dan S (2010). Correlating phosphatidylinositol 3-kinase inhibitor efficacy with signaling pathway status: in silico and biological evaluations. Cancer Res..

[49] Dan S (2002). An integrated database of chemosensitivity to 55 anticancer drugs and gene expression profiles of 39 human cancer cell lines. Cancer Res..

[50] Bridges D (2012). Rab5 proteins regulate activation and localization of target of rapamycin complex 1. J. Biol. Chem..

[51] Matsui T, Fukuda M (2013). Rab12 regulates mTORC1 activity and autophagy through controlling the degradation of amino-acid transporter PAT4. EMBO Rep..

[52] Goldenring JR (2013). A central role for vesicle trafficking in epithelial neoplasia: intracellular highways to carcinogenesis. Nat. Rev. Cancer.

[53] Chia WJ, Tang BL (2009). Emerging roles for Rab family GTPases in human cancer. Biochim. Biophys. Acta.

[54] Engelman JA (2009). Targeting PI3K signalling in cancer: opportunities, challenges and limitations. Nat. Rev. Cancer.

[55] Gills JJ, Dennis PA (2009). Perifosine: update on a novel Akt inhibitor. Curr. Oncol. Rep..

[56] Uchida A, Alami NH, Brown A (2009). Tight functional coupling of kinesin-1A and dynein motors in the bidirectional transport of neurofilaments. Mol. Biol. Cell.

[57] Ying QL (2008). The ground state of embryonic stem cell self-renewal. Nature.

[58] Sakurai T (2016). RNA-binding motif protein 47 inhibits Nrf2 activity to suppress tumor growth in lung adenocarcinoma. Oncogene.

[59] Matsuura K (2011). Identification of a link between Wnt/β-catenin signalling and the cell fusion pathway. Nat. Commun..

[60] Koinuma D (2009). Chromatin immunoprecipitation on microarray analysis of Smad2/3 binding sites reveals roles of ETS1 and TFAP2A in transforming growth factor beta signaling. Mol. Cell. Biol..

[61] Murai F (2015). EZH2 promotes progression of small cell lung cancer by suppressing the TGF-β-Smad-ASCL1 pathway. Cell Discov..

