# Supplementary material for: TUFT1 interacts with RABGAP1 and regulates mTORC1 signaling
Source: Cell Discov. 2018 Jan 9;4:1. doi: 10.1038/s41421-017-0001-2 (PMC5798889; doi:10.1038/s41421-017-0001-2)
Supplement: Supplementary file 1 — Supplementary Information [file 41421_2017_1_MOESM1_ESM.pdf]

Figure S1

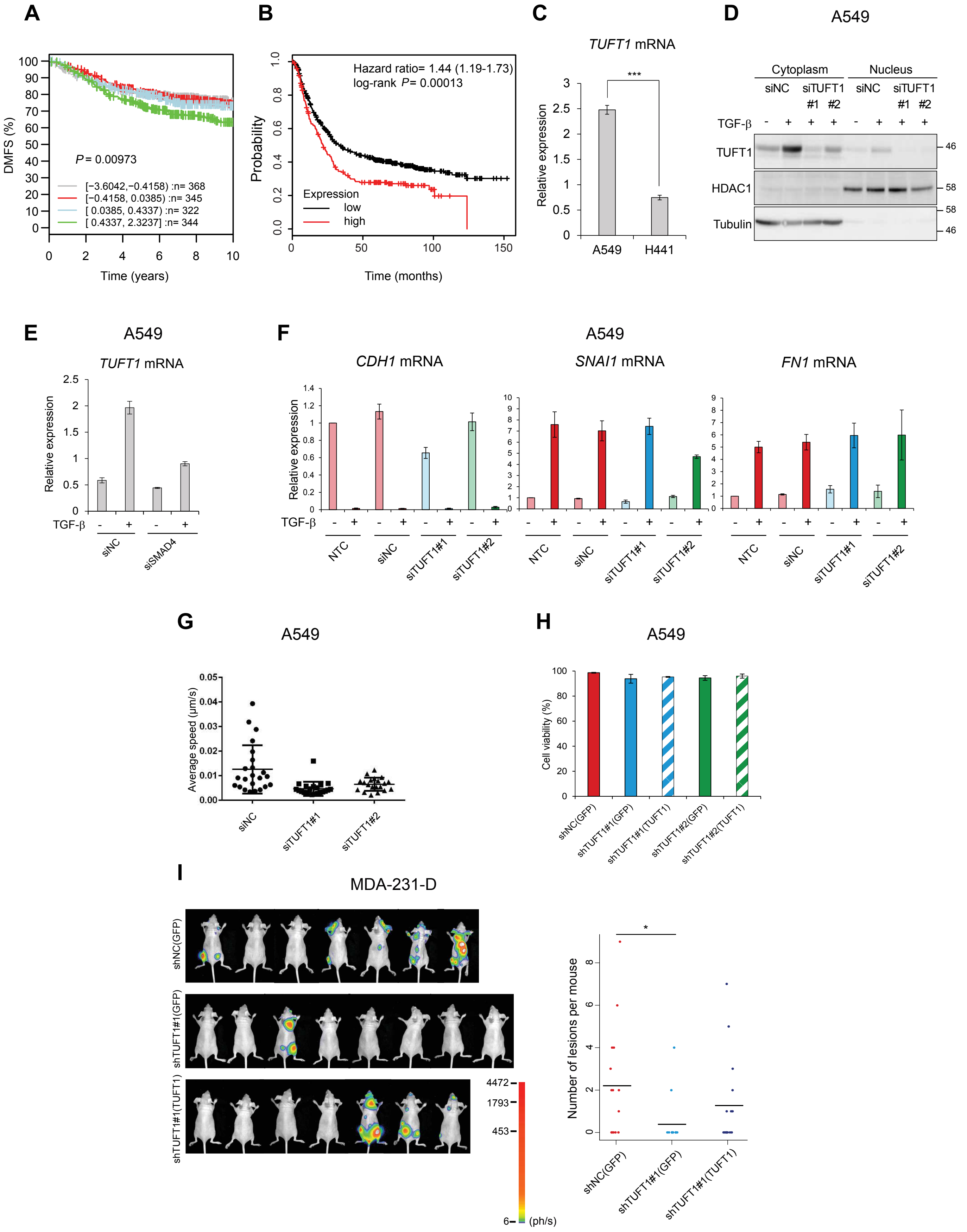
A
B
C
D
A549
TUFT1 mRNA
Cytoplasm
Nucleus
siNC
siTUFT1
#1
#2
TGF-β
TUFT1
HDAC1
Tubulin
DMFS (%)
P= 0.00973
[−3.6042,−0.4158) :n= 368
[−0.4158, 0.0385) :n= 345
[0.0385, 0.4337) :n= 322
[0.4337, 2.3237] :n= 344
Time (years)
Hazard ratio= 1.44 (1.19-1.73)
log-rank P= 0.00013
Probability
Expression
low
high
Time (months)
Relative expression
***
A549
H441
E
F
CDH1 mRNA
SNAI1 mRNA
FN1 mRNA
siSMAD4
NTC
siTUFT1#1
siTUFT1#2
G
H
Average speed (μm/s)
Cell viability (%)
shNC(GFP)
shTUFT1#1(GFP)
shTUFT1#1(TUFT1)
shTUFT1#2(GFP)
shTUFT1#2(TUFT1)
I
MDA-231-D
4472
1793
453
6
(ph/s)
Number of lesions per mouse
*

Figure S1: TUFT1 correlates with poor cancer prognosis and regulates morphological change through an EMT-independent pathway.

(A) Kaplan Meier plot of distant metastasis-free survival (DMFS) of breast cancer patients stratified into quartiles of TUFT1 expression using GOBO meta-analysis.

(B) Kaplan Meier plot of overall survival stratified by median TUFT1 expression using the KM-Plotter version 2015 gastric cancer meta-analysis database.

(C) TUFT1 expression in A549 and NCI-H441 (H441) cells was quantified by qRT-PCR. Results are means ± standard error of the means of three independent experiments. ***$P < 0.001$.

(D) A549 cells were transfected with siRNA for TUFT1 or a negative control siRNA (siNC) in the absence or presence of TGF-β (1 ng/mL) for 48 h. Cell lysates were fractionated into the cytoplasmic and nuclear fractions and immunoblotted. Results are representative of two independent experiments.

(E) Cells were treated as in (D). Induction of TUFT1 was examined by qRT-PCR. Results are means ± standard deviations of two independent experiments.

(F) Cells were treated as in (D). Induction of target genes was examined by qRT-PCR. Expression was compared with no transfection control (NTC). Results are means ± standard error of the means of three independent experiments.

(G) A549 cells expressing indicated shRNAs and GFP were photographed for 4 h using a time-lapse FV10i microscope (Olympus). Average velocity of the cells was calculated by ImageJ.

(H) A549 cells expressing indicated shRNAs and proteins were diluted in Trypan Blue solution. Viability of the cells was calculated by TC10™ Automated Cell Counter (Bio-Rad). Results are means ± standard deviations of two independent experiments.

(I) Metastatic cells were analyzed by in vivo imaging in nude mice injected with MDA-231-D cells (1×10^5 cells) expressing the indicated shRNAs and proteins. The experiment was repeated with similar results, and representative images are shown (left). Dot plot for number of lesions per mouse from the two independent experiments is shown in the right panel. The horizontal bars indicate the mean for each group. N = 15 mice for the shNC(GFP) group, N = 16 mice for the shTUFT1#1(GFP) and shTUFT1#1(TUFT1) groups. *$P < 0.05$. ph/s: photon counts per second.

Figure S2

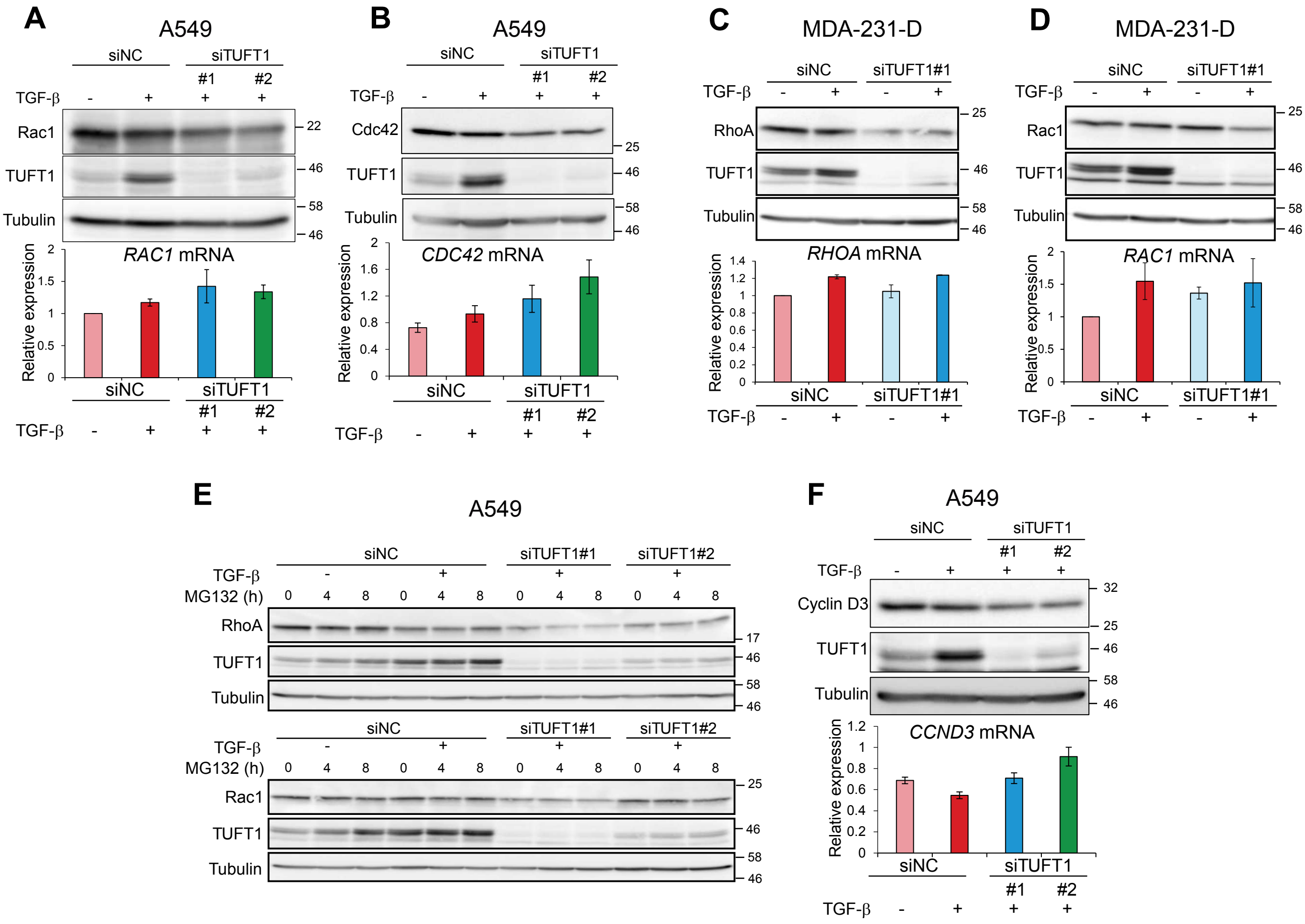

G

NAME	SIZE	NES	FDR q-val
E2F TARGETS	159	4.93	0.000
G2M CHECKPOINT	149	3.84	0.000
MYC TARGETS V1	198	3.82	0.000
MYC TARGETS V2	51	2.31	0.018
MTORC1 SIGNALING	166	2.17	0.017

Figure S2: TUFT1 regulates protein levels of some Rho family proteins and cyclins.

(A, B) A549 cells were transfected with siRNA for TUFT1 or a negative control siRNA (siNC) in the absence or presence of TGF-β (1 ng/mL) for 48 h. Total amounts of Rac1 (A) and Cdc42 (B) were detected by immunoblotting. Results are representative of two independent experiments. mRNA expression was quantified by qRT-PCR (bottom panels). Results are means ± standard error of the means of three independent experiments.

(C, D) MDA-231-D cells were cultured in the absence or presence of TGF-β for 48 h, and total amounts of RhoA (C) and Rac1 (D) were detected by immunoblotting. Results are representative of two independent experiments. mRNA expression was quantified by qRT-PCR (bottom panels). Results are means ± standard error of the means of three independent experiments.

(E) A549 cells were treated as in (A). Cells were then treated with MG132 (10 μM) for the indicated times and harvested for immunoblotting. Results are representative of two independent experiments.

(F) A549 cells were treated as in (A). Total amounts of Cyclin D3 were detected by immunoblotting. Results are representative of two independent experiments. mRNA expression was quantified by qRT-PCR (bottom panel). Results are means ± standard deviations of two independent experiments.

(G) A549 cells were transfected with siTUFT1#1 or siNC. Then, RNA-sequencing and GSEA were performed. The expression cutoff of FPKM (fragments per kilobase of exon per million mapped sequence reads) values ≥10 was applied for evaluation. A list of top-enriched Hallmark gene sets (MSigDB) upregulated in siNC relative to siTUFT1#1 are shown. Size of genes in each set, normalized enrichments score (NES) and false discovery rate (FDR) are shown per each gene set analyzed.

Figure S3

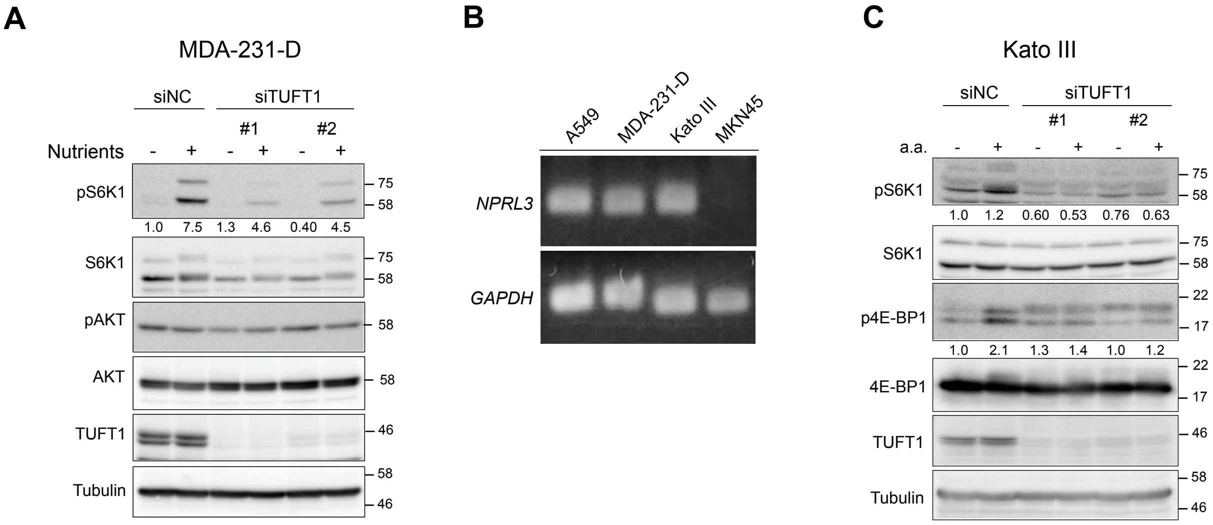

Figure S3: TUFT1 is necessary for mTORC1 activation in a Rag-independent manner.

(A) siRNAs were transfected into MDA-231-D cells. Cells were starved (3 h) or starved and restimulated (10 min) with nutrients. Cell lysates were analyzed by immunoblotting. Results are representative of two independent experiments. Values in the panels in (A) and (C) show the amount of phosphorylated protein relative to the total amount of the protein, which were quantified by ImageJ.

(B) NPRL3 and GAPDH expression was analyzed by RT-PCR in A549, MDA-231-D, Kato III and MKN45 cells. Results are representative of two independent experiments.

(C) siRNAs were transfected into Kato III cells. Cells were starved (3 h) or starved and restimulated (10 min) with amino acids (a.a.). Cell lysates were analyzed by immunoblotting. Results are representative of two independent experiments.

Figure S4

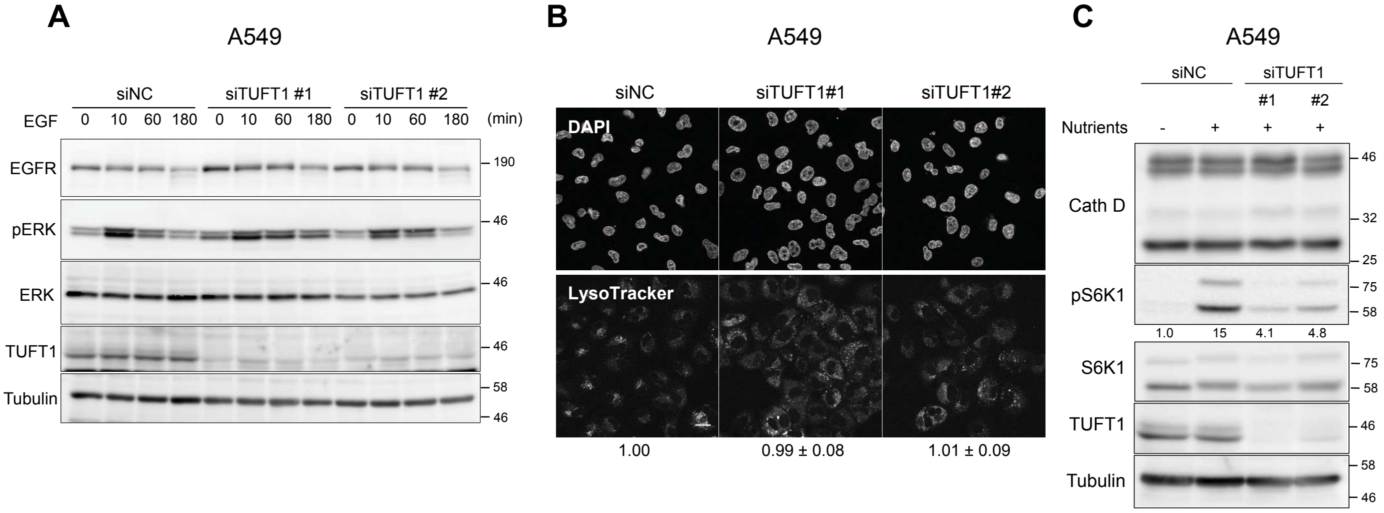

Figure S4: TUFT1 depletion does not affect lysosomal activity.

(A) A549 cells transfected with siRNAs were serum starved for 12 h and treated with EGF (100 ng/mL) for indicated time periods. Cell lysates were harvested for immunoblotting. Results are representative of two independent experiments.

(B) A549 cells were transfected with the indicated siRNAs. Cell were then stained with LysoTracker Red (DND-99) for 1 h. Scale bar, 20 µm. Images stained with DAPI are shown in the upper panels. Results are representative of two independent experiments. Relative signal intensity per cell at each condition was calculated by ImageJ, and shown in the bottom. Images are representative of two independent experiments.

(C) A549 cells were treated with siRNAs. Cells were starved (3 h) or starved and restimulated (10 min) with nutrients. Cell lysates were analyzed by immunoblotting. The effect of TUFT1 depletion on cathepsin D (Cath D) processing from pro-cathepsin D was analyzed. Results are representative of two independent experiments. The relative amount of phosphorylated protein was quantified as in Supplementary Figure S3A.

Figure S5

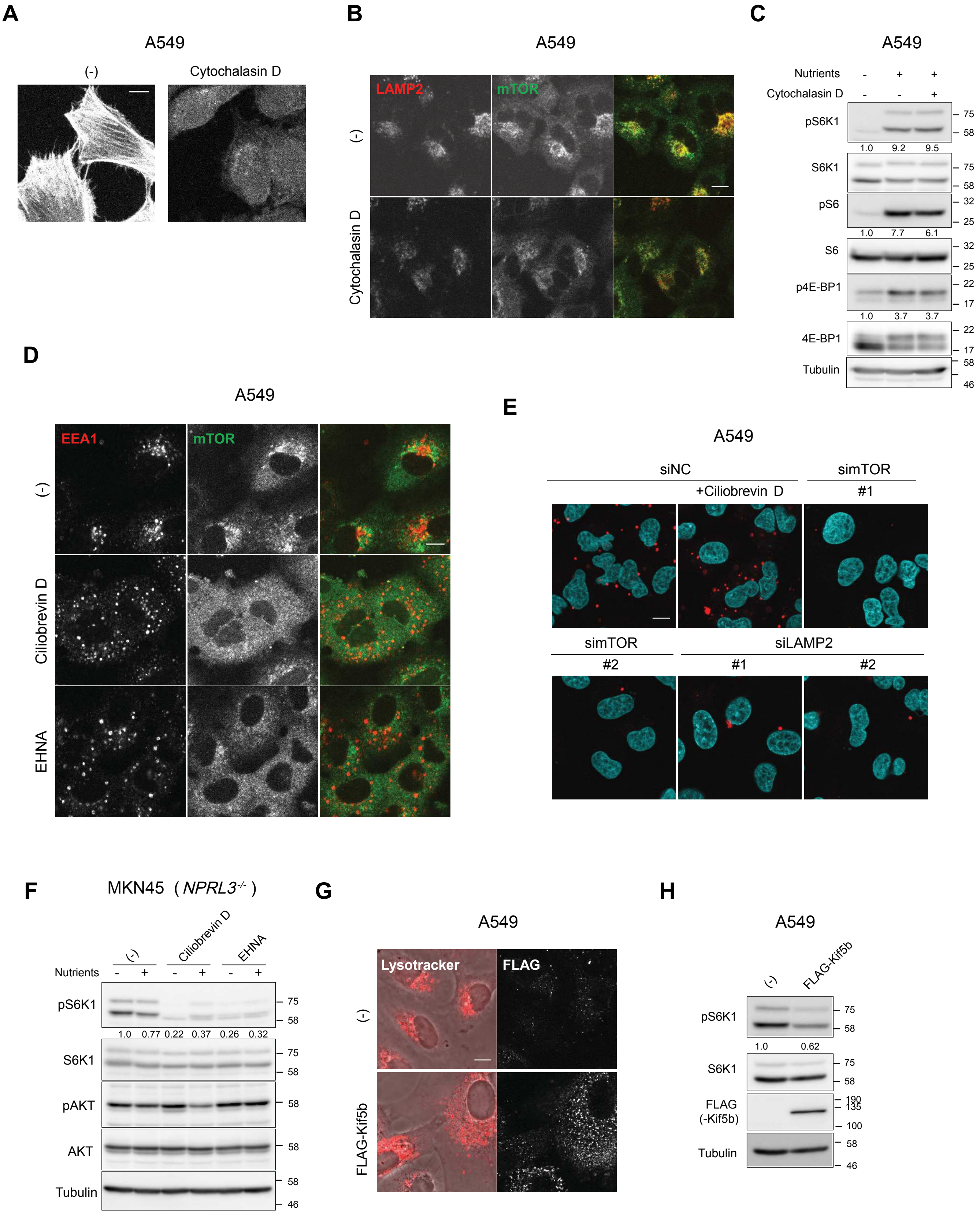

A
A549
(-)
Cytochalasin D
B
A549
LAMP2
mTOR
(-)
Cytochalasin D
C
A549
Nutrients - + +
Cytochalasin D - - +
pS6K1
1.0 9.2 9.5
S6K1
pS6
1.0 7.7 6.1
S6
p4E-BP1
1.0 3.7 3.7
4E-BP1
Tubulin
75 58 75 58 32 25 32 25 22 17 22 17 58 46
D
A549
EEA1
mTOR
(-)
Ciliobrevin D
EHNA
E
A549
siNC
+Ciliobrevin D
simTOR
#1
simTOR
#2
siLAMP2
#1
#2
F
MKN45 (NPRL3-/-)
(-)
Ciliobrevin D
EHNA
Nutrients - + - + - +
pS6K1
1.0 0.77 0.22 0.37 0.26 0.32
S6K1
pAKT
AKT
Tubulin
75 58 75 58 58 58 58 46
G
A549
Lysotracker
FLAG
(-)
FLAG-Kif5b
H
A549
(-)
FLAG-Kif5b
pS6K1
1.0 0.62
S6K1
FLAG
(-Kif5b)
Tubulin
75 58 75 58 190 135 100 58 46

Figure S5: Dynein inhibitors disrupt mTORC1 signaling.

(A) A549 cells were starved (2 h), incubated with cytochalasin D (0.1 µM) for additional 1 h, and then restimulated with nutrients for 10 min. Actin stress fibers were visualized with fluorescein-conjugated phalloidin. Images are representative of two independent experiments. Scale bar, 10 µm.

(B) A549 cells were treated as in (A). Cells were then fixed and immunostained with the indicated antibodies. Images are representative of two independent experiments. Scale bar, 10 µm.

(C) A549 cells were treated as in (A). Cell lysates were collected and subjected to immunoblotting. Results are representative of two independent experiments. The relative amount of phosphorylated protein was quantified in panels (C), (F), and (H), as in Supplementary Figure S3A.

(D) A549 cells were treated with ciliobrevin D (50 µM) or EHNA (500 µM) for 1 h. Cells were then fixed and immunostained with the indicated antibodies. Images are representative of two independent experiments. Scale bar, 10 µm.

(E) A549 cells were treated with the indicated siRNAs. Cells were starved (2 h) and incubated with ciliobrevin D (50 µM) for additional 1 h. Then, the cells were restimulated with nutrients for 10 min. Proximity of mTOR to LAMP2 was detected by in situ PLA. Cell nuclei were counter-stained by DAPI. Images are representative of three independent experiments. Scale bar, 10 µm.

(F) MKN45 cells were starved (2 h) and incubated with ciliobrevin D (50 µM) or EHNA (500 µM) for additional 1 h. Then, the cells were restimulated with nutrients for 10 min. Cell lysates were collected and subjected to immunoblotting. Results are representative of two independent experiments.

(G) A549 cells transfected with indicated plasmids were incubated with LysoTracker Red (DND-99) for 1 h. Images are representative of two independent experiments. Scale bar, 10 µm.

(H) A549 cells transfected with indicated plasmids were lysed and analyzed by immunoblotting.

Figure S6

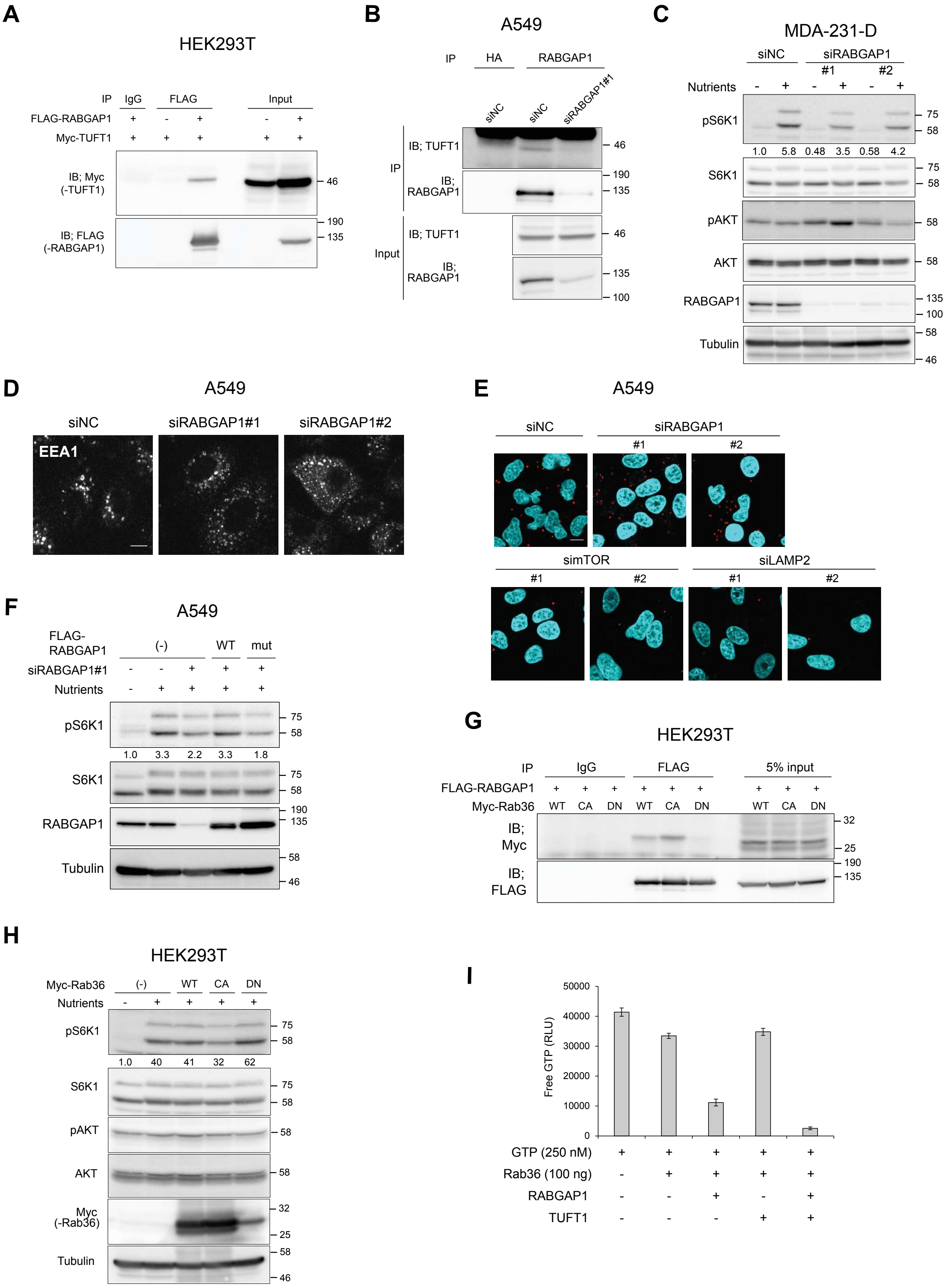
A
HEK293T
IP IgG FLAG Input
FLAG-RABGAP1
Myc-TUFT1
IB; Myc (-TUFT1)
IB; FLAG (-RABGAP1)
B
A549
IP HA RABGAP1
siNC siNC siRABGAP1#1
IB; TUFT1
IB; RABGAP1
Input
C
MDA-231-D
siNC siRABGAP1 #1 #2
Nutrients
pS6K1
1.0 5.8 0.48 3.5 0.58 4.2
S6K1
pAKT
AKT
RABGAP1
Tubulin
D
A549
siNC siRABGAP1#1 siRABGAP1#2
EEA1
E
A549
siNC siRABGAP1 #1 #2
simTOR #1 #2
siLAMP2 #1 #2
F
A549
FLAG-RABGAP1 (-) WT mut
siRABGAP1#1
Nutrients
pS6K1
1.0 3.3 2.2 3.3 1.8
S6K1
RABGAP1
Tubulin
G
HEK293T
IP IgG FLAG 5% input
FLAG-RABGAP1
Myc-Rab36 WT CA DN WT CA DN WT CA DN
IB; Myc
IB; FLAG
H
HEK293T
Myc-Rab36 (-) WT CA DN
Nutrients
pS6K1
1.0 40 41 32 62
S6K1
pAKT
AKT
Myc (-Rab36)
Tubulin
I
Free GTP (RLU)
GTP (250 nM)
Rab36 (100 ng)
RABGAP1
TUFT1

Figure S6: RABGAP1 regulates intracellular compartment positioning, vesicular trafficking and mTORC1 signaling.

(A) HEK293T cells were transfected with expression plasmids for RABGAP1 and TUFT1. Cell lysates were subjected to immunoprecipitation using an antibody against FLAG. Immunoprecipitates and 5% input extracts were immunoblotted with the indicated antibodies. Results are representative of two independent experiments.

(B) Lysates of A549 cells transfected with the indicated siRNAs were subjected to immunoprecipitation using the indicated antibodies. Immunoprecipitates and 3% input extracts were immunoblotted with the indicated antibodies. Results are representative of two independent experiments.

(C) MDA-231-D cells were treated with the indicated siRNAs. Cells were starved (3 h) or starved and restimulated (10 min) with nutrients. Cell lysates were analyzed by immunoblotting. Results are representative of two independent experiments. The relative amount of phosphorylated protein was quantified in panels (C), (F), and (H), as in Supplementary Figure S3A.

(D) A549 cells were transfected with siRNAs, and cells were starved (3 h) and restimulated (10 min) with nutrients. Cells were fixed and immunostained with an antibody against EEA1. Images are representative of two independent experiments. Scale bar, 10 μm.

(E) A549 cells were treated as in (D). Proximity of mTOR to LAMP2 was detected by in situ PLA using antibodies against mTOR and LAMP2. Cell nuclei were counter-stained with DAPI. Images are representative of three independent experiments. Scale bar, 10 μm.

(F) A549 cells stably expressing indicated proteins were transfected with siRNAs (siNC (-) or siRABGAP1#1) and treated as in (C). Cell lysates were analyzed by immunoblotting. Results are representative of two independent experiments. WT: wild-type, mut: GAP activity-deficient mutant.

(G) HEK293T cells were transfected with the indicated expression plasmids. Cell lysates were subjected to immunoprecipitation using an antibody against FLAG. Immunoprecipitates and 5 % input extracts were immunoblotted with the indicated antibodies. Results are representative of two independent experiments. WT: wild-type, CA: constitutively active form, DN: dominant negative form.

(H) HEK293T cells transfected with the indicated plasmids were treated as in (C). Results are representative of two independent experiments. WT: wild-type, CA: constitutively active form, DN: dominant negative form.

(I) HEK293T cells expressing FLAG-RABGAP1 or FLAG-TUFT1 were lysed and immunoprecipitated with anti-FLAG antibody. Immunoprecipitated beads were incubated with or without recombinant Rab36 (#ab160571, abcam). In vitro GTPase reaction was performed using in vitro GAP activity assay kit (GTPase-Glo Assay, Promega) in accordance with the manufacturer's instructions. Results are means ± standard deviations of three independent experiments. RLU; Relative Light Unit.

Figure S7

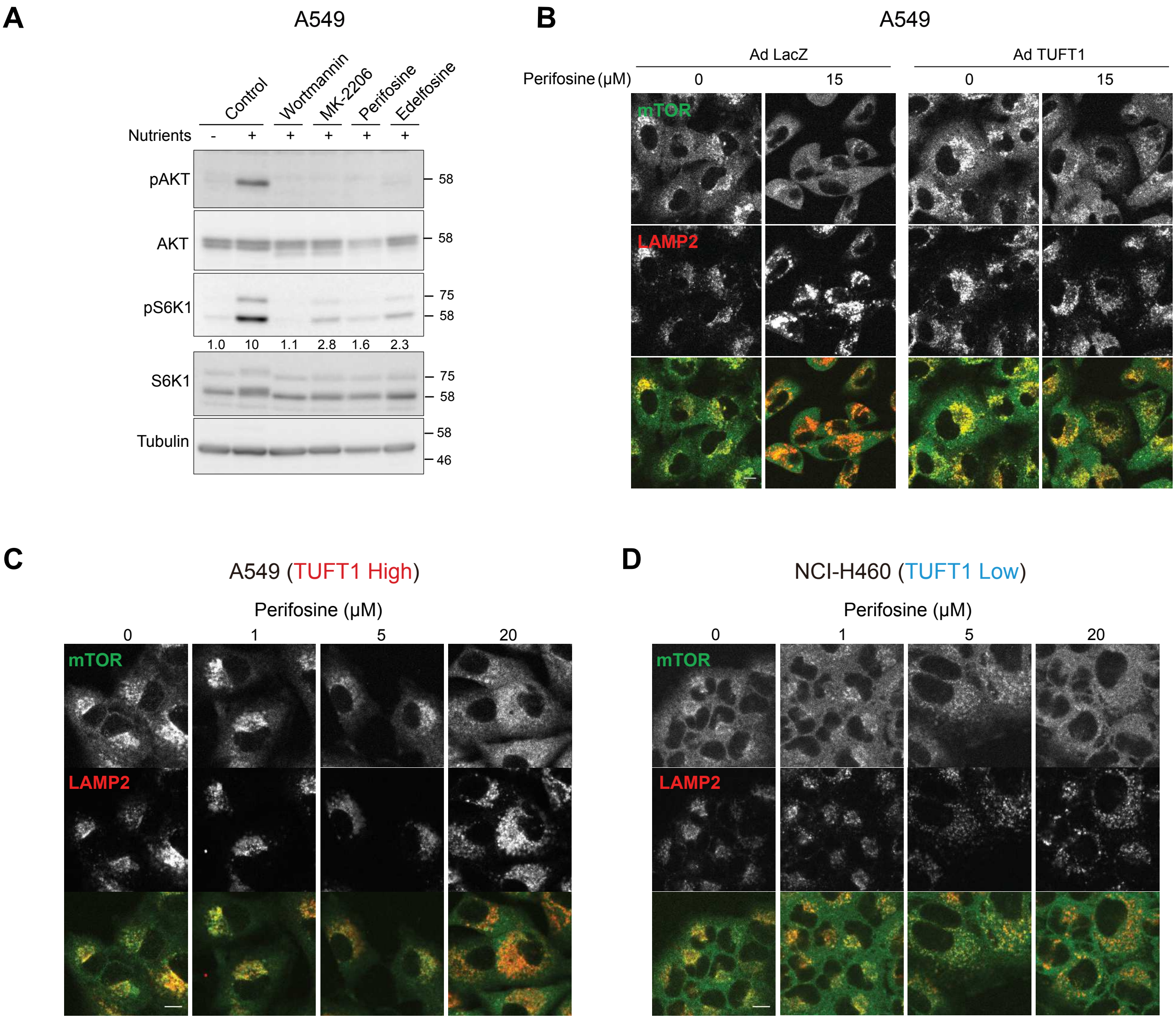

Figure S7: TUFT1 expression positively correlates with perifosine resistance.
(A) A549 cells were starved (3 h) in the presence of the indicated inhibitors or starved and restimulated (10 min) with nutrients. The concentrations of the inhibitors used were: wortmannin (2 μM), MK-2206 (2 μM), perifosine (20 μM) or edelfosine (10 μM). Cell lysates were then immunoblotted. Results are representative of two independent experiments. The relative amount of phosphorylated protein was quantified as in Supplementary Figure S3A.
(B) A549 cells adenovirally-transduced with LacZ or TUFT1 were starved (3 h) in the presence of the indicated inhibitors and restimulated (10 min) with nutrients. Cells were fixed and immunostained with the indicated antibodies. Merged figures are shown in the bottom. Scale bar, 10 μm. Images are representative of two independent experiments.
(C, D) A549 (C) and NCI-H460 (D) cells were starved (3 h) in the presence of perifosine (0-20 μM) and restimulated with nutrients (10 min). Cells were then fixed and stained with the indicated antibodies. Both figures are merged and shown in the bottom. Scale bars, 10 μm. Images are representative of two independent experiments.

Supplementary Table S1. Results of drug sensitivity test in the PI3K-AKT-mTOR pathway.

**compounds**	**average |$\log_{10}$ GI50|**	**Pearson r**	***P* value**
Perifosine	5.075	-0.64612	0.000272
Akt Inhibitor III	4.720	-0.49821	0.008175
Methotrexate	6.249	-0.4754	0.012206
Akt inhibitor II	5.064	-0.38932	0.044726
6-Mercaptopurine	5.132	-0.338	0.084642
(-)-Deguelin	5.191	-0.31364	0.11114
Vinblastine	8.622	-0.28731	0.146198
Akt Inhibitor X	5.427	-0.28025	0.156815
Topotecan	6.964	-0.24954	0.209378
Akt Inhibitor VIII	5.581	-0.24707	0.214077
Bleomycin	4.844	-0.23393	0.240229
Navelbine	8.441	-0.22955	0.249394
Carboplatin	4.060	-0.21789	0.274911
Cytarabine	4.978	-0.21195	0.288547
NVP-BEZ235	8.051	-0.20213	0.311986
Aclacinon	6.956	0.201754	0.312897
AZD6244	4.825	-0.19743	0.323601
SN-38	7.467	-0.19448	0.33103
Actinomycin-D	8.810	-0.19089	0.34021
PI3K-alpha inhibitor IV	6.100	-0.19023	0.341908
4-Hydroperoxycyclophosphamide	4.993	-0.18845	0.346534
6-Thioguanine	5.857	-0.18591	0.353172
Akt Inhibitor XI	4.978	-0.18155	0.364783
Vincristine	8.111	-0.17948	0.370366
E7010	6.402	-0.16007	0.425128
Etoposide	5.207	-0.15466	0.44115
5-Fluorouracil	4.583	-0.15253	0.44754
Gemcitabine	6.542	-0.14699	0.464386
Akt Inhibitor V	4.616	0.135241	0.501213
Akt inhibitor	4.877	-0.12225	0.543544
E7070	4.870	-0.12023	0.550283
Cl-F-ara-A	5.626	-0.10057	0.617703
PI103	6.801	-0.09989	0.620095
FMDC	6.029	-0.09812	0.626342

ZSTK474	6.321	-0.0977	0.6278
Temsirolimus	6.665	-0.09607	0.633605
Akt Inhibitor IX	5.991	0.094799	0.638111
Pirarubicin	8.042	0.074832	0.710666
PX-866	5.753	-0.07214	0.720664
Carboquone	7.031	-0.07157	0.722785
LY294002	5.078	0.060414	0.764682
Doxorubicin	7.018	-0.05834	0.77255
Rapamycin	6.621	-0.05133	0.799301
GDC-0941	6.285	-0.05081	0.801278
Mitomycin-C	5.905	-0.04918	0.807525
Neocarzinostatin	6.459	0.048664	0.809523
PD98059	4.115	0.046192	0.819036
Oxaliplatin	5.238	-0.04433	0.826207
Epirubicin	7.096	0.041956	0.835398
wortmannin	4.965	0.041939	0.835462
U0126	4.906	0.038305	0.849552
Akt Inhibitor IV	6.559	-0.03769	0.851925
HCFU	4.839	-0.03662	0.856086
IC87114	4.082	0.033607	0.867832
Paclitaxel	7.597	0.033426	0.868541
RAD-001	7.441	-0.02849	0.887823
ICRF-193	4.372	-0.02442	0.903769
TGX221	4.964	0.023338	0.908013
Docetaxel	7.798	0.022492	0.911334
Amsacrine	6.187	0.018903	0.925438
AS605240	5.008	-0.01531	0.93957
Cisplatin	5.120	0.009597	0.962107
CPT-11	4.890	-0.0069	0.972746
Melphalan	4.672	-0.00519	0.979493
Semaxanib	4.294	0.004729	0.981325
Nedaplatin	4.642	0.001674	0.99339
Mitoxantrone HCl	7.163	-0.00038	0.998504

Supplementary Table S2. Sequences of the primers used for qRT-PCR.

Gene	Forward primers (5'-3')	Reverse primers (5'-3')
TUFT1	TTGCTAGGGATGGAGACGGA	TTGGCAGTCAGCATTGTTGC
RHOA	TATCGAGGTGGATGGAAAGC	TTCTGGGGTCCACTTTTCTG
RAC1	AACCAATGCATTTCCTGGAGA	CTGTTTGCGGATAGGATAGGG
CDC42	GTGTGTTGTTGTGGGCGATG	TGTGGATAACTCAGCGGTCG
CDH1	TGCACCAACCCTCATGAGTG	GTCAGTATCAGCCGCTTTCAG
FN1	GACAGGAGGAAATAGCCC	CATCGTGCAAGGCAACCAC
SNAI1	TTCTCACTGCCATGGAATTCC	GCAGAGGACACAGAACCAGAAA
CCND1	ATCTACACCGACAACTCCATCC	GCATTTTGGAGAGGAAGTGTTC
CCND3	TGACCATCGAAAAACTGTGC	ACAGAGGGCCAAAAAGGTCT
GAPDH	TCTTTTGCGTCGCCAGCCGAG	TGACCAGGCGCCCAATACGAC
NPRL3	CTGAGGACCTCCGCATGTTTG	CAAACAGCATGAGCAGCTGG
